# Introgression of Chinese haplotypes contributed to the improvement of Danish Duroc pigs

**DOI:** 10.1111/eva.12716

**Published:** 2018-12-13

**Authors:** Minhui Chen, Guosheng Su, Jinluan Fu, Aiguo Wang, Jian‐Feng Liu, Mogens S. Lund, Bernt Guldbrandtsen

**Affiliations:** ^1^ Department of Molecular Biology and Genetics, Centre for Quantitative Genetics and Genomics Aarhus University Tjele Denmark; ^2^ Department of Animal Genetics, Breeding and Reproduction China Agricultural University Beijing China

**Keywords:** genomics, introgression, pigs, population genetics

## Abstract

The distribution of Asian ancestry in the genome of Danish Duroc pigs was investigated using whole‐genome sequencing data from European wild boars, Danish Duroc, Chinese Meishan and Bamaxiang pigs. Asian haplotypes deriving from Meishan and Bamaxiang occur widely across the genome. Signatures of selection on Asian haplotypes are common in the genome, but few of these haplotypes have been fixed. By defining 50‐kb windows with more than 50% Chinese ancestry, which did not exhibit extreme genetic differentiation between Meishan and Bamaxiang as candidate regions, the enrichment of quantitative trait loci in candidate regions supports that Asian haplotypes under selection play an important role in contributing genetic variation underlying production, reproduction, meat and carcass, and exterior traits. Gene annotation of regions with the highest proportion of Chinese ancestry revealed genes of biological interest, such as *NR6A1*. Further haplotype clustering analysis suggested that a haplotype of Chinese origin around the *NR6A1* gene was introduced to Europe and then underwent a selective sweep in European pigs. Besides, functional genes in candidate regions, such as *AHR* and *PGRMC2*, associated with fertility, and *SAL1*, associated with meat quality, were identified. Our results demonstrate the contribution of Asian haplotypes to the genomes of European pigs. Findings herein facilitate further genomic studies such as genomewide association study and genomic prediction by providing ancestry information of variants.

## 1 INTRODUCTION

Adaptive introgression is a process whereby beneficial variants are acquired by introgression from related species or populations and are then spread further in the recipient population by positive selection. Since introgression can be an important source of advantageous alleles and the initial frequencies of beneficial alleles can be high if genetic admixture is intense (Hedrick, 2013; Jordan, 2016), introgression has the potential to speed adaptive evolution. Indeed, recent studies have provided evidence of adaptive evolution of introgressed haplotypes in humans (Huerta‐Sanchez et al., 2014; Juric, Aeschbacher, & Coop, 2015), plants (Hufford et al., 2013) and animals (Eriksson et al., 2008; Figueiro et al., 2017; Pardo‐Diaz et al., 2012).

Domestication of pigs has occurred independently from wild boars in Europe and Asia (Giuffra et al., 2000; Larson et al., 2005). It is well documented that around 1,700 Chinese pigs were introduced into Northern Europe to improve local breeds to meet the demand of intensified agriculture (White, 2011). A genomic study of Isla del Coco feral pigs, a population derived from British pigs and isolated since 1793, showed evidence of crossbreeding with Chinese pigs, demonstrating crossing as early as the 18th century (Bianco, Soto, Vargas, & Perez‐Enciso, 2015). A previous study (Chen et al., 2017) confirmed that most European pig breeds contained about 20% ancestry from Chinese pigs. Artificial selection on introgressed Chinese haplotypes has further been demonstrated in European Large White pigs (Bosse et al., 2014). Besides, adaptive introgression in pigs has also been observed on the X chromosome (Ai et al., 2015).

To identify introgressed haplotypes accurately, extant groups genetically similar to the source populations of admixture should be sampled. In our previous study (Chen et al., 2017), we found that the main source of the introgression from China to Europe was pigs from South China, which were genetically close to Bamaxiang. To better characterize the hybrid nature of European pig genome, in this study, we used both Bamaxiang pigs from South China and Meishan pigs from East China to identify the introgressed Chinese haplotypes in the Danish Duroc pigs.

The aim of the study was to detect selection on introgressed Chinese haplotypes and evaluate their contribution to the improvement of European pigs. To achieve this goal, we carried out a study utilizing whole‐genome sequencing data from 16 European wild boars (EUW), 90 Danish Duroc, 30 Meishan and 6 Bamaxiang pigs. EUW, Meishan and Bamaxiang pigs were used as donors to paint the genomes of Duroc pigs using ChromoPainter (Lawson, Hellenthal, Myers, & Falush, 2012), a recently developed software for local ancestry inference.

## 2 METHODS

### 2.1 Sample collection and DNA preparation

Samples from 30 Meishan pigs and 90 Danish Duroc pigs were collected. For Meishan pigs, genomic DNA was extracted from ear tissue using a standard phenol–chloroform method; for Duroc pigs, genomic DNA was extracted from blood sample using the QIAsymphony DNA Mini Kit (Qiagen). Sequencing was performed on the Illumina HiSeq 2000 platform. Whole‐genome sequencing data from 16 EUW (Bosse et al., 2014; Frantz et al., 2015) and six Bamaxiang pigs (Ai et al., 2015) were obtained. For EUW, FASTQ files for seven individuals were downloaded from European Nucleotide Archive (ENA, accession PRJEB9922), and BAM files for nine individuals were downloaded from ENA (accession ERP001813). For Bamaxiang pigs, we downloaded FASTQ files from NCBI Sequence Read Archive (accession SRA096093).

### 2.2 SNP calling and filtering

For each individual, the read‐pairs were pre‐processed using PRINSEQ (Schmieder & Edwards, 2011). Reads were trimmed to a minimum base PHRED quality of 20 from the 3′‐end and removed if shorter than 51 bp, with more than 3 Ns, or a mean quality score <18. Filtered reads were aligned to the porcine reference genome build 10.2 (Groenen et al., 2012) by the Burrows–Wheeler Aligner (BWA version 0.7.17) (Li & Durbin, 2009), employing the "mem" algorithm. SAMtools (version 1.8) (Li et al., 2009) was used for sorting, merging and removing potential PCR duplications. Variants were identified in all samples simultaneously using the Genome Analysis Toolkit's (GATK version 3.5) UnifiedGenotyper (McKenna et al., 2010) with default parameter values, which included downsampling of SNP sites with more than 250 reads per sample. We used vcftools (version 0.1.15) (Danecek et al., 2011) to retain only SNPs. Finally, we identified 40,116,524 SNPs in 142 genomes. As introgression differs between sex chromosomes and autosomes, we only retained SNPs on autosomes for subsequent analyses.

### 2.3 Population structure analyses

Population structure analyses were conducted together with our previously merged SNP array data from Eurasian domestic pigs and wild boars (Chen et al., 2017). These array data were collected from multiple studies (Ai, Huang, & Ren, 2013; Chen et al., 2017; Goedbloed et al., 2013; Manunza et al., 2013; Wilkinson et al., 2013), involving 695 individuals and 30,549 autosomal SNPs. Therefore, we extracted from the sequence data to obtain genotypes at all variants present in the merged data set. To reduce the effect of uneven sample size per population (McVean, 2009), we randomly selected six individuals for SNP array genotyped populations with more than six individuals, while keeping all sequenced individuals. We performed PCA (principal component analysis) using EIGENSOFT 6.0.1 (Patterson, Price, & Reich, 2006) and population structure analysis by ADMIXTURE (version 1.23) (Alexander, Novembre, & Lange, 2009) for *K* = 1 to 20 ancestral populations using default options.

### 2.4 Chromosome painting using ChromoPainter

Chromosome painting is a method to characterize shared ancestry between individuals that takes linkage disequilibrium (LD) information into account. We reconstructed haplotypes and imputed missing genotypes using BEAGLE (version 4.1) (Browning & Browning, 2007). Only bi‐allelic SNPs were used for chromosome painting. We used ChromoPainter v2 (Lawson et al., 2012) to perform chromosome painting on Duroc individuals using EUW, Meishan and Bamaxiang pigs as donor populations. We ran ChromoPainter twice, as recommended in the user manual. In the first run, we used all donor individuals to paint nine randomly selected Duroc individuals on six randomly selected chromosomes (SSC 2, 5, 7, 10, 11, 16 and 17). The aim of this run was to estimate switch rate, global mutation rate and copying probabilities by running 50 iterations of the expectation–maximization algorithm. We averaged estimated values of each parameter across these chromosomes, weighting by the number of SNPs, and then across individuals, to obtain estimates of parameters. In the second run, we used the estimates from the first run to perform chromosome painting for all individuals and autosomes. As a result, we obtained an estimated ancestry proportion for each SNP in the genome of Duroc.

To evaluate the variation of ancestry proportion, we divided the genome into non‐overlapping 50‐kb windows. In each window, we separately estimated the average proportion of ancestry from Meishan, Bamaxiang and EUW across SNPs. We focused our analyses on windows with a high proportion of introgression from Chinese pigs, rather than windows with a significant signal of selection. Specifically, we selected windows with more than 50% Chinese ancestry (the sum of Meishan and Bamaxiang ancestries) as candidate regions of adaptive introgression. To avoid the confounding of introgression from European pigs to China, we further excluded windows with extreme *F*_ST_ between Meishan and Bamaxiang, *that is*, windows with *F*_ST_ falling in the top 5% of the empirical distribution.

### 2.5 *F*_ST_ calculation

To quantify genomic differentiation, we calculated *F*_ST_ values between Duroc and Meishan, Duroc and Bamaxiang, and Meishan and Bamaxiang. Hudson's *F*_ST_ (Hudson, Boos, & Kaplan, 1992) was calculated for each bi‐allelic SNP using the “PopGenome” package (Pfeifer, Wittelsburger, Ramos‐Onsins, & Lercher, 2014) in R. Then, we averaged *F*_ST_ scores across SNPs within non‐overlapping 50‐kb windows. Because the range of true *F*_ST_ values by definition is between 0 and 1 (Wright, 1951), we set *F*_ST_ to 0 for windows with negative average estimates of *F*_ST_. The differentiation between Duroc and Chinese pigs was calculated as the average of *F*_ST_ between Duroc and Meishan, and *F*_ST_ between Duroc and Bamaxiang.

### 2.6 Gene annotation and functional analyses

We conducted gene annotation on five regions with the highest proportions of Meishan ancestry and five regions with the highest proportions of Bamaxiang ancestry. We took the following approaches to define these regions using Meishan ancestry as an example. We first selected 50‐kb windows with the highest proportions of Meishan ancestry, which did not exhibit extreme *F*_ST_ between Meishan and Bamaxiang (i.e., exclude windows with *F*_ST_ higher than 95% quantile of the empirical distribution); then, these windows were extended in both directions until the next window with <50% Meishan ancestry or with extreme *F*_ST_ between Meishan and Bamaxiang. Since long regions were more likely to be caused by selection, we chose the five regions with at least two windows. Gene contents and QTL (quantitative trait locus) numbers in these regions were retrieved from the Ensembl Genes 89 Database using BioMart (Kinsella et al., 2011) and from the Animal QTL Database (Hu, Park, & Reecy, 2016).

We counted the number of QTLs in Animal QTL Database (Hu et al., 2016) which overlapped with candidate regions with more than 50% Chinese ancestry without extreme *F*_ST_ between Meishan and Bamaxiang. First, 25,610 QTLs were extracted from the database. Among these, 8,937 have been identified by linkage analysis. These were excluded due to uncertain genomic locations. The remaining 16,673 QTLs, identified by association studies, were retained for further analysis. These QTLs were classified into five groups corresponding to five classes of traits as defined in the Animal QTL Database (Hu et al., 2016): meat and carcass, production, reproduction, health and exterior traits. We excluded 817 QTLs not located on autosomes or spanning more than 1 Mb. A total of 15,856 QTLs remained, with 7,654, 957, 837, 4,774 and 1,634 associated with meat and carcass, production, reproduction, health and exterior traits. The length of those QTL ranged from 40 bp to 1 Mb, with a mean of 24 ± 115 kb (mean ± *SD*). Of note, the database defined QTL regions as follows: (a) when flanking SNPs were given, the region was defined by the actual locations; (b) when only the centre SNP was given, the region was defined as a small region to create a minimum visible map window in a genome browser (the middle of the region was the actual location of centre SNP); (c) when SNPs were completely missing, the region was estimated from linkage association. We defined the mid‐point of a QTL region as the peak position. Finally, we counted the number of QTLs whose peak positions were located within candidate regions. When two QTL records associated with traits in the same trait group and had the same genomic interval in the Animal QTL Database, they were counted as one QTL. The same method was applied to each trait group separately.

To test for enrichment among all QTLs and among trait‐specific QTLs within candidate regions, we applied a permutation test. We concatenated the autosomes into a circular genome, in the order from SSC1 to SSC18. A permuted sample was formed by moving candidate regions along the genome by a randomly chosen amount. This permutation did not change the relative positions between these windows preserving their correlation structure. We then computed the number of all QTLs and those belonging to each trait group that presented in these simulated windows using the same method as above. In total, 10,000 permutations were performed. The distribution of numbers of QTLs observed in the permutated regions was treated as the null distribution from which we computed the significance levels of the number of QTLs observed in the real data.

### 2.7 Haplotype clustering in NR6A1

To check whether the NR6A1 haplotype in Danish Duroc pigs was introgressed from Meishan pigs or a breed genetically close to Meishan, we compared haplotypes within a reference pool of more Chinese pig breeds. We further collected whole‐genome sequencing data from Chinese pigs, including five Erhualian (Ai et al., 2015), two Jinhua (Frantz et al., 2015), four Tongcheng (Wang et al., 2015), six Luchuan (Ai et al., 2015) and six Wuzhishan pigs (Ai et al., 2015). For Meishan and Duroc, we randomly selected 10 individuals from each population. We used the same procedure as above to call variants in the 2‐Mb region (298–300 Mb on SSC1) around the NR6A1 gene. BEAGLE (version 4.1) (Browning & Browning, 2007) was used to impute missing genotypes and to phase haplotypes. Haplostrips (Marnetto, Huerta‐Sánchez, & Price, 2017) was used to cluster haplotypes in the peak region 299.3–299.4 Mb (including two windows with more than 90% Meishan ancestry from ChromoPainter analysis) and haplotype in the region 299.05–299.45 Mb (including eight windows with more than 50% Meishan ancestry from ChromoPainter analysis, regardless of *F*_ST_ between Meishan and Bamaxiang). We used Duroc as the reference population, and calculated the distance of other haplotypes from the reference as the number of SNPs with different alleles. Similar haplotypes were clustered together and ordered based on the distance to the reference.

## 3 RESULTS

### 3.1 Population structure

**Figure 1:**
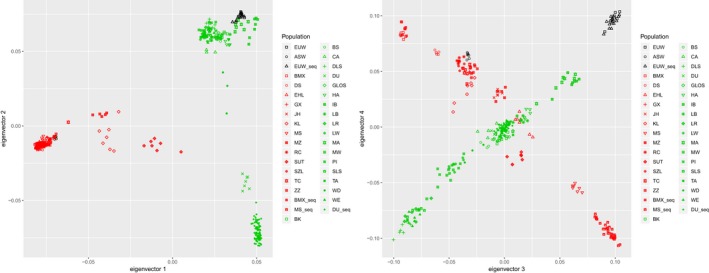
The plot of principal component analysis on Eurasian domestic pigs and wild boars. Genotyped wild boars are coloured with black; Chinese domestic pigs are coloured with red; European domestic pigs are coloured with green. ASW: Asian wild boars; BK: Berkshire; BMX: Bamaxiang; BMX_seq: sequenced Bamaxiang; BS: British Saddleback; CA: Canarian; DLS: Danish Landrace; DS: Dongshan; DU: Duroc; DU_seq: sequenced Duroc; EHL: Erhualian; EUW_seq: sequenced EUW; GLOS: Gloucestershire Old Spots; GX: Ganxi; HA: Hampshire; IB: Ibérico; JH: Jinhua; KL: Kele; LB: Large Black; LR: Landrace; LW: Large White; MA: Mangalica; MS: Meishan; MS_seq: sequenced Meishan; MW: Middle White; MZ: Min; PI: Pietrain; RC: Rongchang; SLS: Pied Landrace; SUT: Sutai; SZL: Shaziling; TA: Tamworth; TC: Tongcheng; WD: White Duroc; WE: Welsh; ZZ: Tibetan

**Figure 2:**
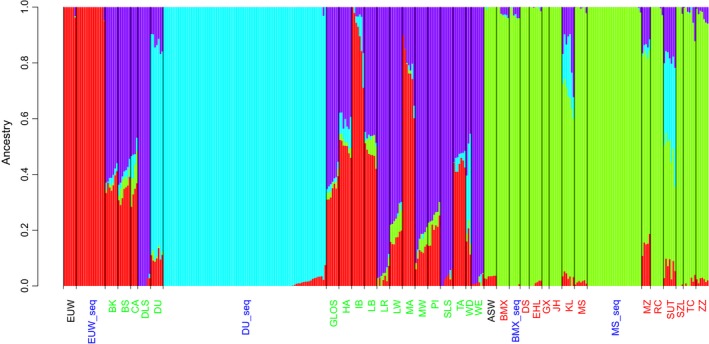
Ancestries of each sample using four ancestral populations (*K* = 4) in the ADMIXTURE analysis. Genotyped wild boars are labelled with black; genotyped Chinese domestic pigs are labelled with red; genotyped European domestic pigs are labelled with green; sequenced wild boars and domestic pigs are labelled with blue. ASW: Asian wild boars; BK: Berkshire; BMX: Bamaxiang; BMX_seq: sequenced Bamaxiang; BS: British Saddleback; CA: Canarian; DLS: Danish Landrace; DS: Dongshan; DU: Duroc; DU_seq: sequenced Duroc; EHL: Erhualian; EUW_seq: sequenced EUW; GLOS: Gloucestershire Old Spots; GX: Ganxi; HA: Hampshire; IB: Ibérico; JH: Jinhua; KL: Kele; LB: Large Black; LR: Landrace; LW: Large White; MA: Mangalica; MS: Meishan; MS_seq: sequenced Meishan; MW: Middle White; MZ: Min; PI: Pietrain; RC: Rongchang; SLS: Pied Landrace; SUT: Sutai; SZL: Shaziling; TA: Tamworth; TC: Tongcheng; WD: White Duroc; WE: Welsh; ZZ: Tibetan

The first four components of PCA are shown in Figure 1. The first principal component separated Chinese pigs from European pigs; the second principal component separated Duroc pigs from other European pigs; the third and fourth principal components separated populations into subgroups. As expected, sequenced individuals clustered together with corresponding genotyped individuals from the same breed. These results indicated that sequenced individuals are representative for their populations. ADMIXTURE analysis supported this result. The cross‐validation error dropped slowly after *K* reached 10, and was minimized at *K = *19 (Supporting Information Figure S1). To evaluate the genetic similarity between sequenced individuals and genotyped individuals, we examined ADMIXTURE results for *K* = 4 (Figure 2). ADMIXTURE identified four components corresponding to 1: Duroc, 2: EUD (European domestic pigs) except Duroc, 3: EUW and 4: Chinese wild boars and domestic pigs. These four components could reveal whether there was recent gene flow to our sequenced individuals. The ancestry composition was similar in sequenced individuals and genotyped individuals from the same breed. However, one EUW individual (Sample ID: WB44U06) had about 5% of EUD ancestry, and was removed from further analysis. The remaining individuals of EUW, Meishan and Bamaxiang pigs contained little ancestry from other components.

### 3.2 Chromosome painting

**Figure 3:**
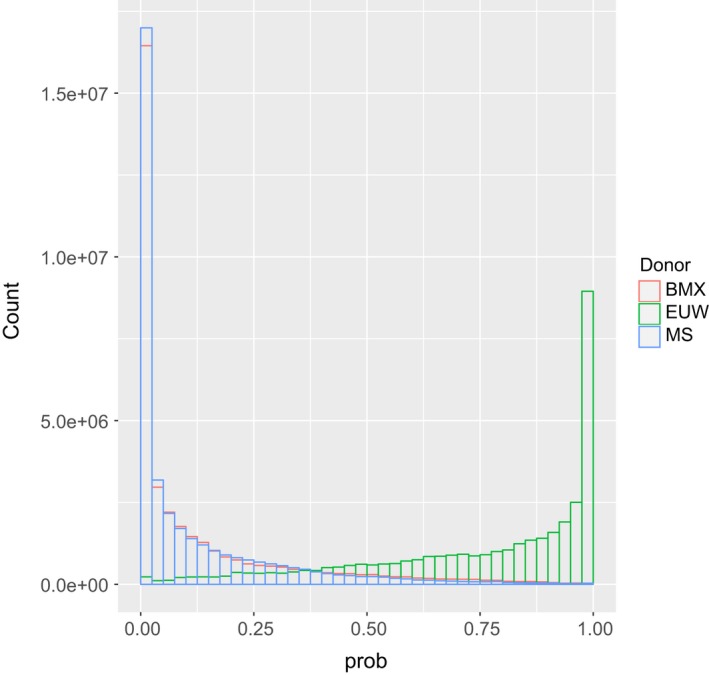
The distribution of ancestry proportion across SNPs in the genome

The first run of ChromoPainter (Lawson et al., 2012) arrived at final values of *N*_e_ = 3,106 for switch rate, 0.015 for global mutation rate, and 0.14, 0.13 and 0.73 for probabilities of copying from Meishan, Bamaxiang and EUW. In the second run of ChromoPainter, we estimated the proportion of ancestry from Meishan, Bamaxiang and EUW for each SNP in the Duroc genome. Figure 3 shows the distribution of proportions of ancestries contributed by Meishan, Bamaxiang, and EUW across the genome. As expected, most SNPs were fixed or were approaching fixation for variants derived from EUW. Besides, Meishan ancestry and Bamaxiang ancestry had almost the same distribution, confirming our previous result that pigs from East China also contributed to the introgression from Chinese pigs to European ones (Chen et al., 2017). The average proportions of ancestries over non‐overlapping 50‐kb windows are shown in Supporting Information Figure S2. Ancestry proportions varied a lot along the genome, with a genomic average of 0.12 ± 2.90e‐5 (mean ± *SE*), 0.13 ± 3.27e‐5 and 0.75 ± 4.28e‐5 from Meishan, Bamaxiang and EUW, respectively. Out of 48,555 windows, 8,086 had more than 50% ancestry from Chinese pigs (sum of Meishan and Bamaxiang ancestries) and *F*_ST_ between Meishan and Bamaxiang lower than 95% quantile (*F*_ST_ = 0.51) of the empirical distribution (details in Supporting Information Table S1). Among these, 1,640 windows had more than 50% Meishan ancestry, and 2,630 windows had more than 50% Bamaxiang ancestry. These results suggest selection for Chinese‐derived haplotypes in the Duroc genome.

### 3.3 Population differentiation

*F*_ST_ values were calculated to measure the degree of genetic differentiation between Duroc and Chinese pigs. Supporting Information Figure S2 shows the distribution of *F*_ST_ values averaged over all SNPs within non‐overlapping 50‐kb windows. Regions with a high proportion of ancestry from Chinese pigs presented low *F*_ST_ values. The correlation between 50‐kb window‐based *F*_ST_ value and proportion of Chinese ancestry was *r* = −0.58 (*p*‐value <2.2e‐16). The strong correlation confirmed the accuracy of local ancestry inference. In windows with more than 70% Meishan ancestry, the average of 50‐kb window‐based *F*_ST_ between Duroc and Meishan was 0.33 ± 0.18 (mean ± *SD*), much lower than the value (average *F*_ST_ 0.45 ± 0.17) between Duroc and Bamaxiang. This demonstrates the presence of Meishan ancestry in Duroc.

### 3.4 Gene annotation and functional analyses

Gene annotation of five regions with the highest proportion of Meishan ancestry and five regions with the highest proportions of Bamaxiang ancestry is shown in Table S2. In total, these ten regions covered 4.1 Mb. All of the 10 regions contained genes that were annotated as known or novel genes. The 32.4–33.75 Mb region on SSC 7 with 94% Chinese Meishan ancestry contained the highest number of QTLs, in total 282 QTLs. These QTLs associated with a variety of traits, such as meat quality and carcass, production and exterior traits.

**Figure 4:**
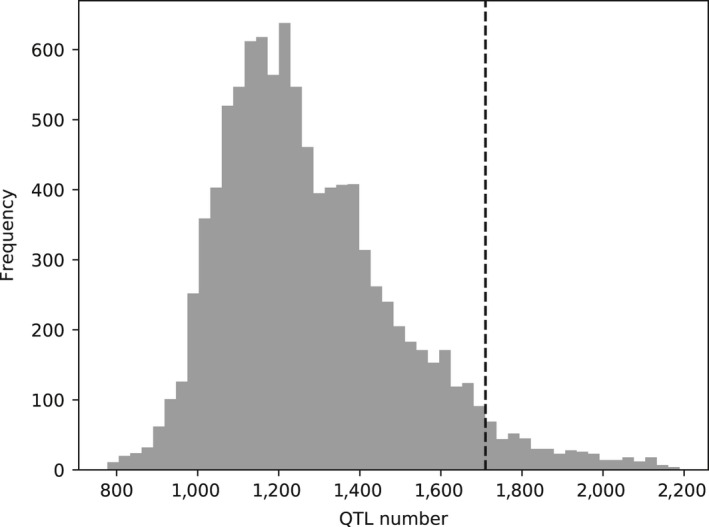
The number of QTLs in 50‐kb windows with more than 50% Chinese ancestry. The histogram is the null distribution of QTL numbers simulated by permutation test. The dashed line indicates the observed number of QTLs

**Figure 5:**
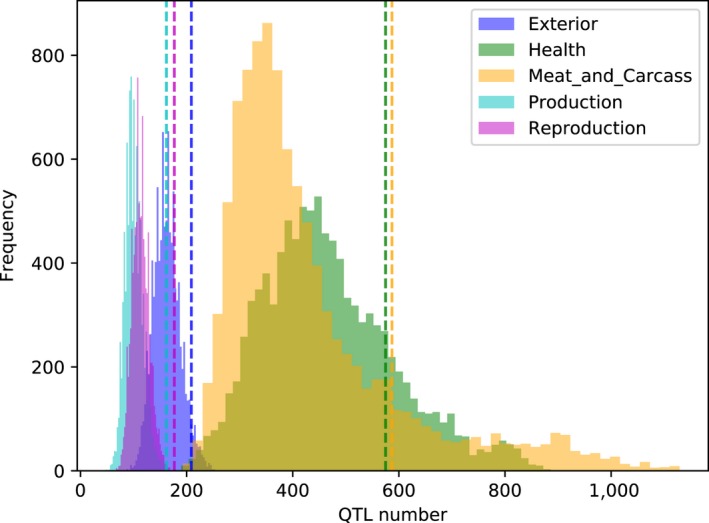
The number of QTLs associated with meat and carcass, production, reproduction, health, and exterior traits in 50‐kb windows with more than 50% Chinese ancestry. The histograms are the null distributions of number of QTLs associated with each trait group by permutation test. The dashed line indicate the observed number of QTLs associated with each trait group

In order to identify potentially selected traits, QTLs located within candidate regions (windows with more than 50% Chinese ancestry and *F*_ST_ lower than 95% quantile of empirical distribution) were counted. The 1,710 QTLs within these windows included 587, 162, 177, 575 and 209 of them associated with meat and carcass, production, reproduction, health and exterior traits, respectively. Figures 4 and 5 compare the observed number of QTLs with the null distribution simulated by a permutation test. As shown in Figure 4, the combined number of QTLs associated with all traits was significantly enriched (*p* = 0.0455). Figure 5 shows the number of QTLs counting for each trait group separately. QTLs associated with meat and carcass, exterior, production and reproduction were significantly enriched in candidate regions with *p* values of 0.0230, 0.0445, 0.0008 and 0.0002.

### 3.5 Introgression of *NR6A1* haplotype

**Figure 6:**
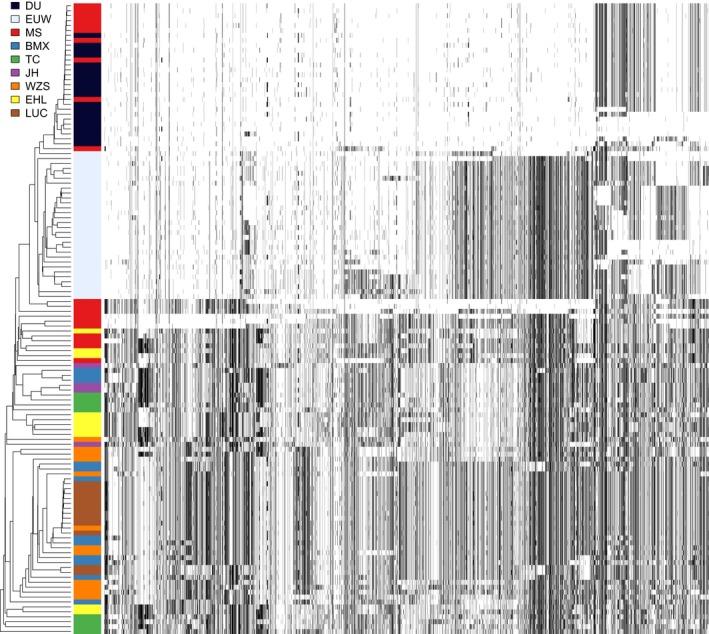
Haplotype clustering on NR6A1 locus (299.05–299.45 Mb on SSC1). Haplotypes are clustered and sorted by increasing distance to the Duroc reference population. Each row represents one haplotype, and each column represents one SNP. The colours in the left column of the panel indicate to which population the haplotypes belong. Reference alleles are represented as white spots, and alternative alleles are represented as black spots. BMX: Bamaxiang; DU: Duroc; EHL: Erhualian; EUW: European wild boars; JH: Jinhua; LUC: Luchuan; MS: Meishan; TC: Tongcheng; WZS: Wuzhishan

Figure 6 showed the result of haplotype clustering in the region 299.05–299.45 Mb on SSC1. Duroc and EUW haplotypes formed a subgroup of Chinese pigs, with some Meishan haplotypes dispersing among those of Duroc. This result suggested that *NR6A1* haplotypes in Duroc were introgressed from Meishan pigs. In the region 299.3–299.4 Mb (Supporting Information Figure S3), Duroc and a portion of Meishan haplotypes clustered together and formed an outgroup of EUW and Chinese pigs. This result confirmed that the *NR6A1* haplotypes in Duroc were genetically closer to those in Meishan pigs, while the direction of introgression was not clear. And haplotypes in Meishan and Duroc might originate from a third population, which was genetically distinct from European and Chinese pigs.

## 4 DISCUSSION

In this study, we used ChromoPainter (Lawson et al., 2012) to analyse the genome of Danish Duroc to infer local ancestry by using EUW, Meishan pigs and Bamaxiang pigs as donor populations. Our results revealed a large number of genomic regions with a high level of introgression of Asian haplotypes. It was suggested that pigs from both South and East China contributed to the introgression from Chinese pigs into European ones. Meishan and Bamaxiang pigs can reveal the majority of introgressed Asian haplotypes in the genome of European pigs. However, of note, since Meishan and Bamaxiang might not be the precise direct source of introgression to European pigs, it could result in the loss of Asian haplotypes which are genetically distinct from Meishan and Bamaxiang.

Some regions with introgression from Chinese pigs were highly significantly enriched in QTLs affecting production and reproduction. These introgressed haplotypes were assumed to improve the performance of European pigs. This can have two (mutually non‐exclusive) causes: First, haplotypes from Chinese pigs may harbour alleles conveying superior performance for these traits, reflecting superior performance of Chinese pigs at the time of introgression; and two, pigs have been selected for traits that generally tend to produce QTLs that have not been fixed in the population. However, there is limited evidence to reflect selective pressure on Chinese haplotypes to improve health traits. It may be because these traits have been difficult to measure and have historically not been under strong selection. However, the QTLs analysed here are derived from different pig breeds and lines. As a result, some of them might not remain associated with traits in Danish Duroc. Further studies using QTLs from Danish Duroc population are needed to confirm the selection on introgressed haplotypes.

The 10 regions with the highest proportions of ancestry from Meishan or Bamaxiang overlapped hundreds of QTLs and genes of potential interest. Of particular interest is the region 299.3–299.45 Mb on SSC 1, which contains two protein‐coding genes including the *NR6A1* gene. This introgression from Meishan into Danish Duroc is further proved in the result of haplotype clustering that Duroc and Meishan haplotypes cluster together and locate as a subgroup of Chinese pigs (Figure 6). However, in the result of the region 299.3–299.4 Mb (Supporting Information Figure S3), Meishan and Duroc haplotypes locate as an outgroup of EUW and Chinese pigs. One possible reason might be that there is not enough variation in this region to correctly place Meishan and Duroc cluster in the tree. This gene has been extensively investigated and is considered a causal gene affecting the number of vertebrae (Mikawa et al., 2005, 2007 ). There is also evidence showing that this gene underwent a selective sweep in European commercial and local breeds (Rubin et al., 2012). In our study, the high haplotype homozygosity in Duroc haplotypes (as shown in Figure 6) also confirms the selection on this haplotype. These results suggest that the *NR6A1* haplotype from Meishan or a closely related breed was introduced into European breeds and subsequently fixed due to selective breeding for large body size.

We here identified a list of genes where the introgressed Chinese haplotypes have spread extensively in Duroc pigs. Many of these have effects on traits under selection. For example, there is more than 99% Chinese ancestry in the 95.5‐ to 95.55‐Mb window on SSC 9, containing the *AHR* gene, the only one gene in this window. This gene plays an important role in the female reproductive system (Hernandez‐Ochoa, Karman, & Flaws, 2009). Selection on *AHR* haplotypes of China origin has also been reported in European Large White pigs (Bosse et al., 2014). Examples of positively selected Chinese haplotypes, that were reported previously and confirmed in this study, include genes associated with fertility (*PGRMC2*: Bosse et al., 2014; with 62% Chinese ancestry), meat quality (*SAL1*: Bosse et al., 2014; with 72% Chinese ancestry), fat deposition (*FASN*: Bosse et al., 2015; with 55% Chinese ancestry), growth (*BMP3*: Bosse et al., 2015; with 52% Chinese ancestry) and pigmentation (*RAB38*: Bosse et al., 2015; with 53% Chinese ancestry). In contrast, we confirmed relatively low proportions of Chinese ancestry for several genes as previously observed, such as *MC1R* (Bosse et al., 2015; Fang, Larson, Soares Ribeiro, Li, & Andersson, 2009; with 4% Chinese ancestry), *KIT* (Bosse et al., 2015; with <1% Chinese ancestry) and *MBNL1* (Bosse et al., 2015; with 19% Chinese ancestry). These results suggest that in these cases, Chinese haplotypes were not positively selected.

## 5 CONCLUSION

Admixture during the Industrial Revolution of Chinese pigs imported into Europe to improve the performance of European breeds has contributed a substantial fraction of the genomes of European domesticated pigs. This introgression is highly uneven across the genome, and that introgressed regions are associated with traits that have been actively selected for in the direction that would favour haplotypes of Asian origin.

## CONFLICT OF INTEREST

None declared.

## DATA ACCESSIBILITY

Sequencing data from Meishan pigs have been deposited in the NCBI Short Read Archive (SRA) (project accession no. PRJNA378496). Chromosome painting results are available from the Dryad Digital Repository: https://doi.org/10.5061/dryad.vh6t0c7.
