# Supplementary figures and images for: Introgression of Chinese haplotypes contributed to the improvement of Danish Duroc pigs
Source: Evol Appl. 2018 Dec 13;12(2):292–300. doi: 10.1111/eva.12716 (PMC6346729; doi:10.1111/eva.12716)

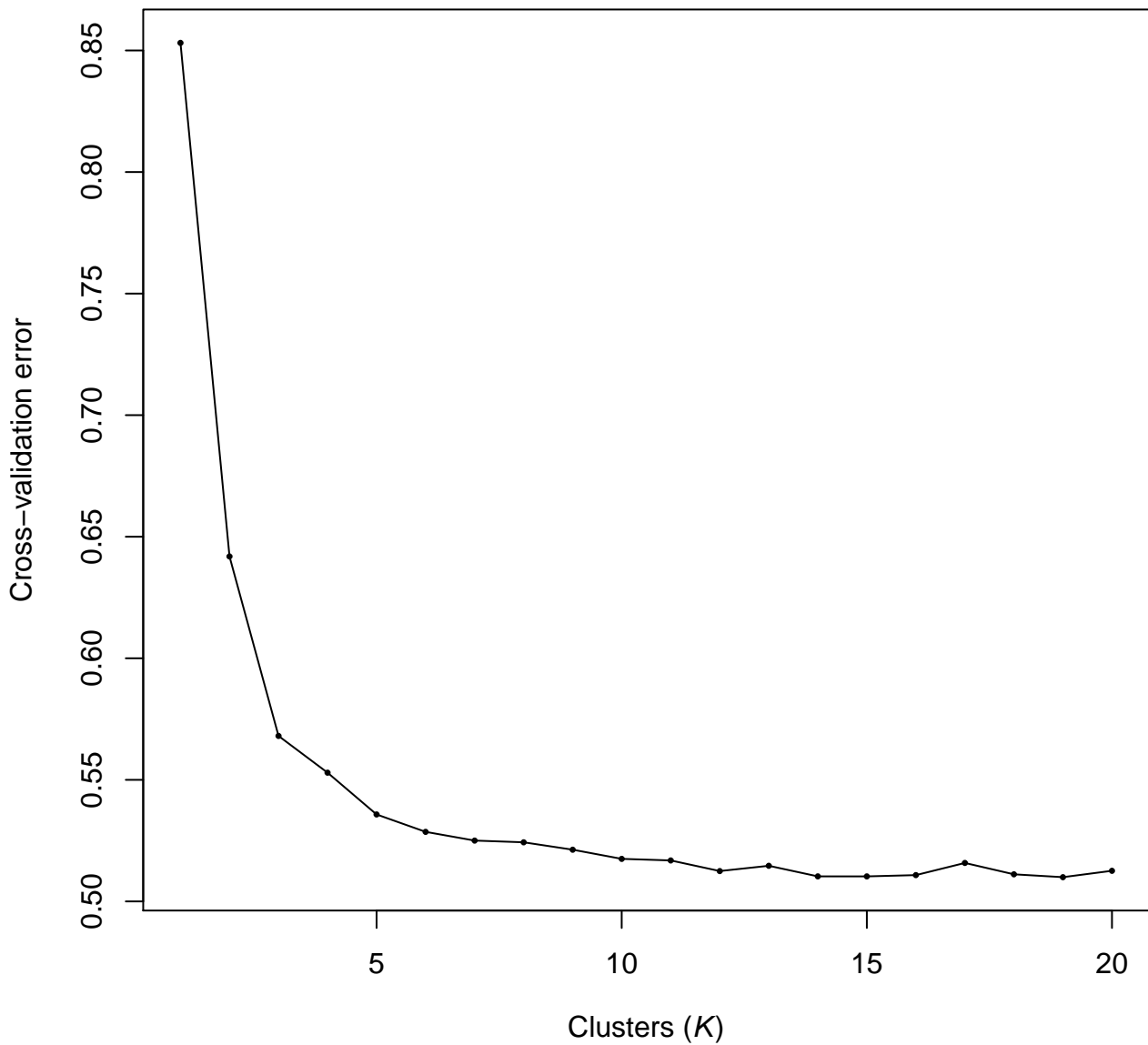

Supplement: Supplementary file 1 [file EVA-12-292-s001.pdf]

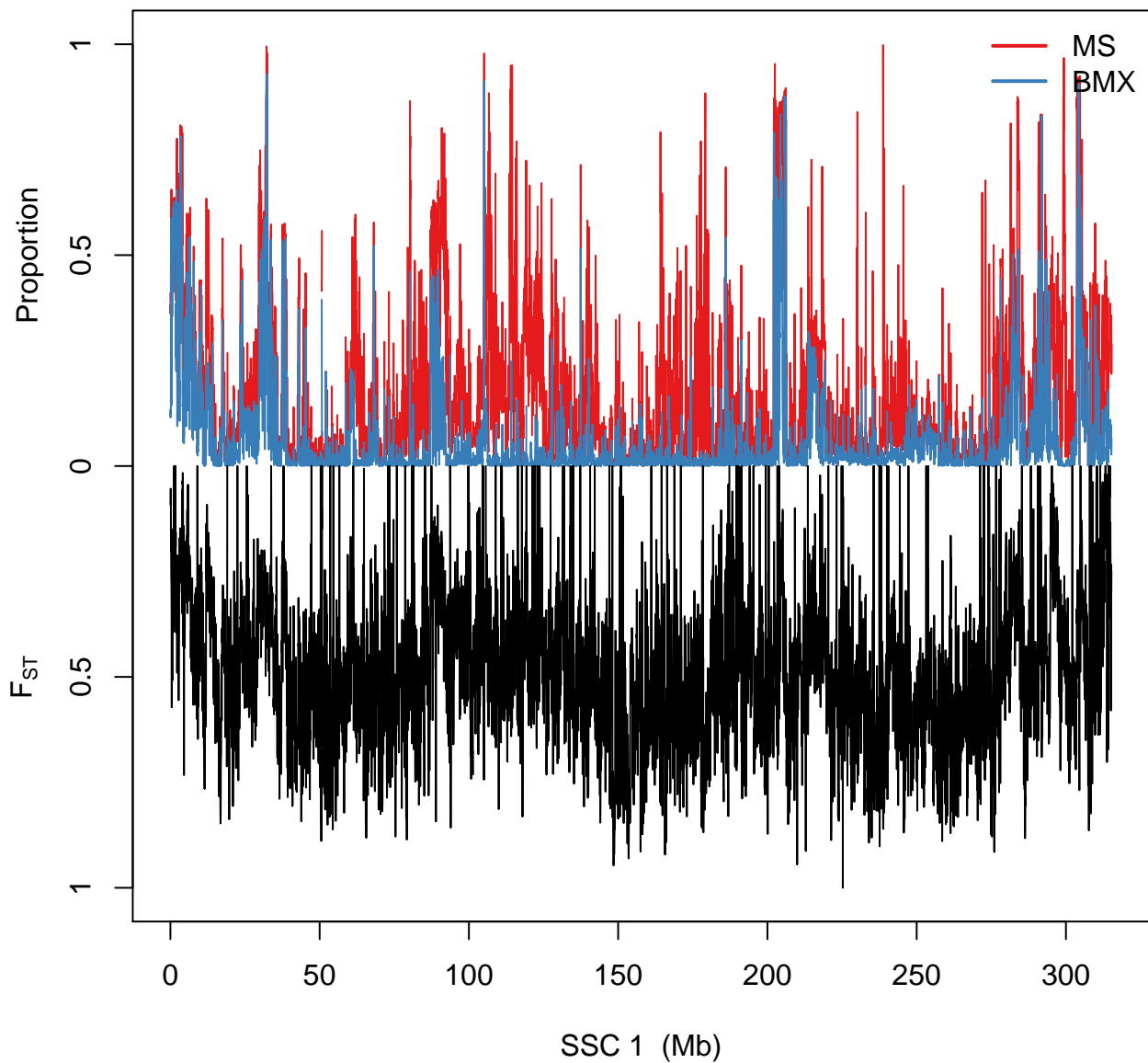

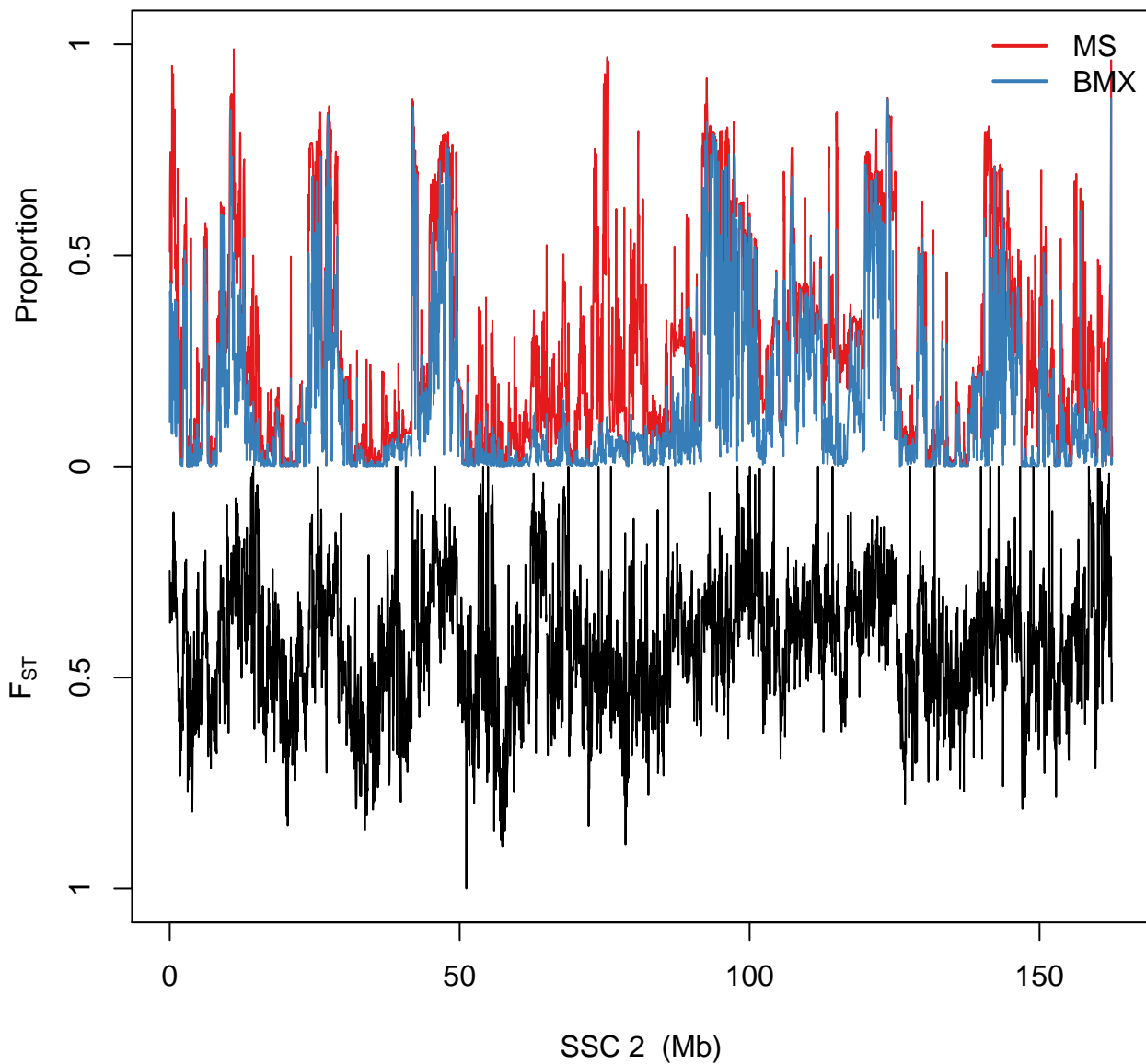

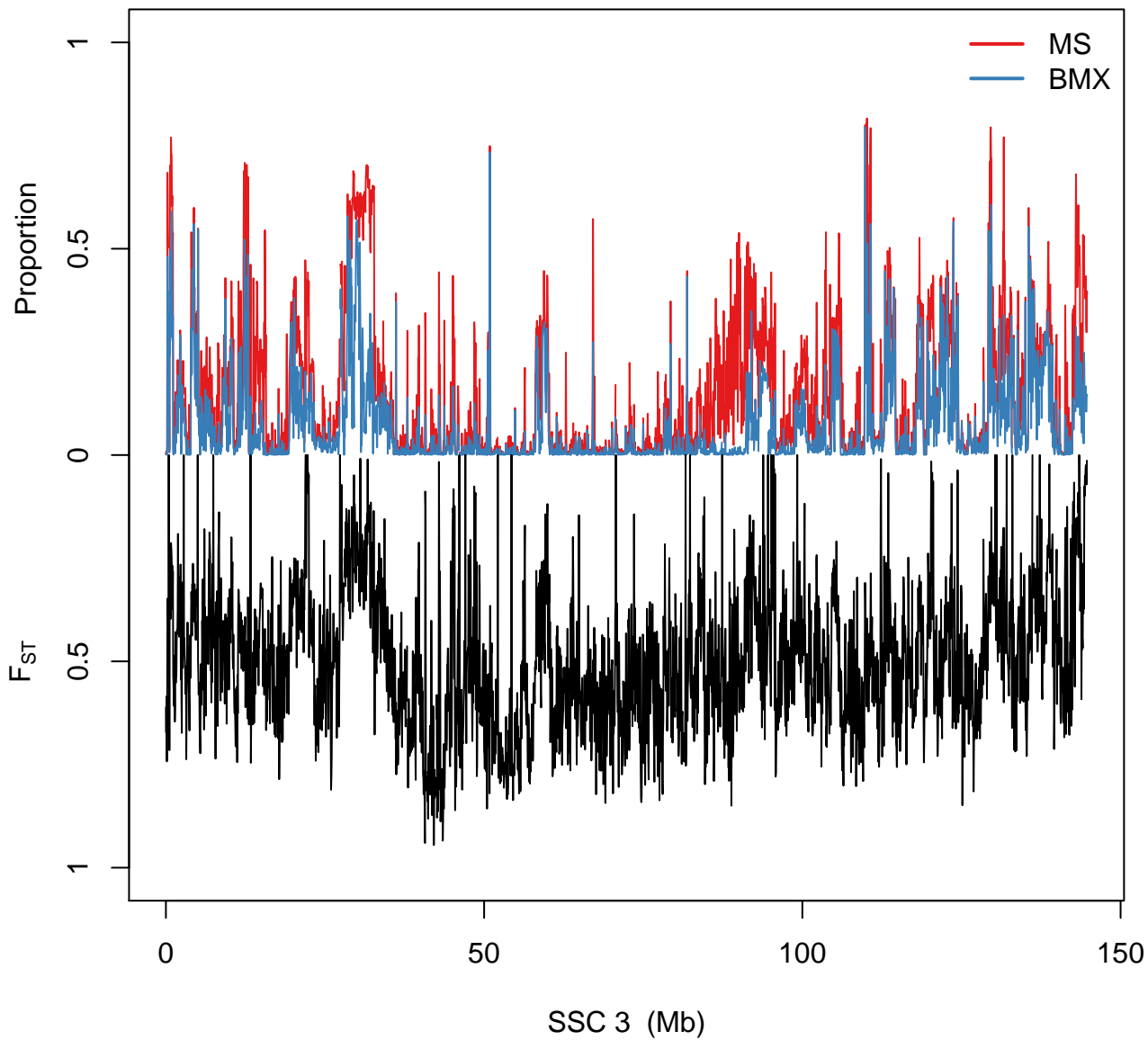

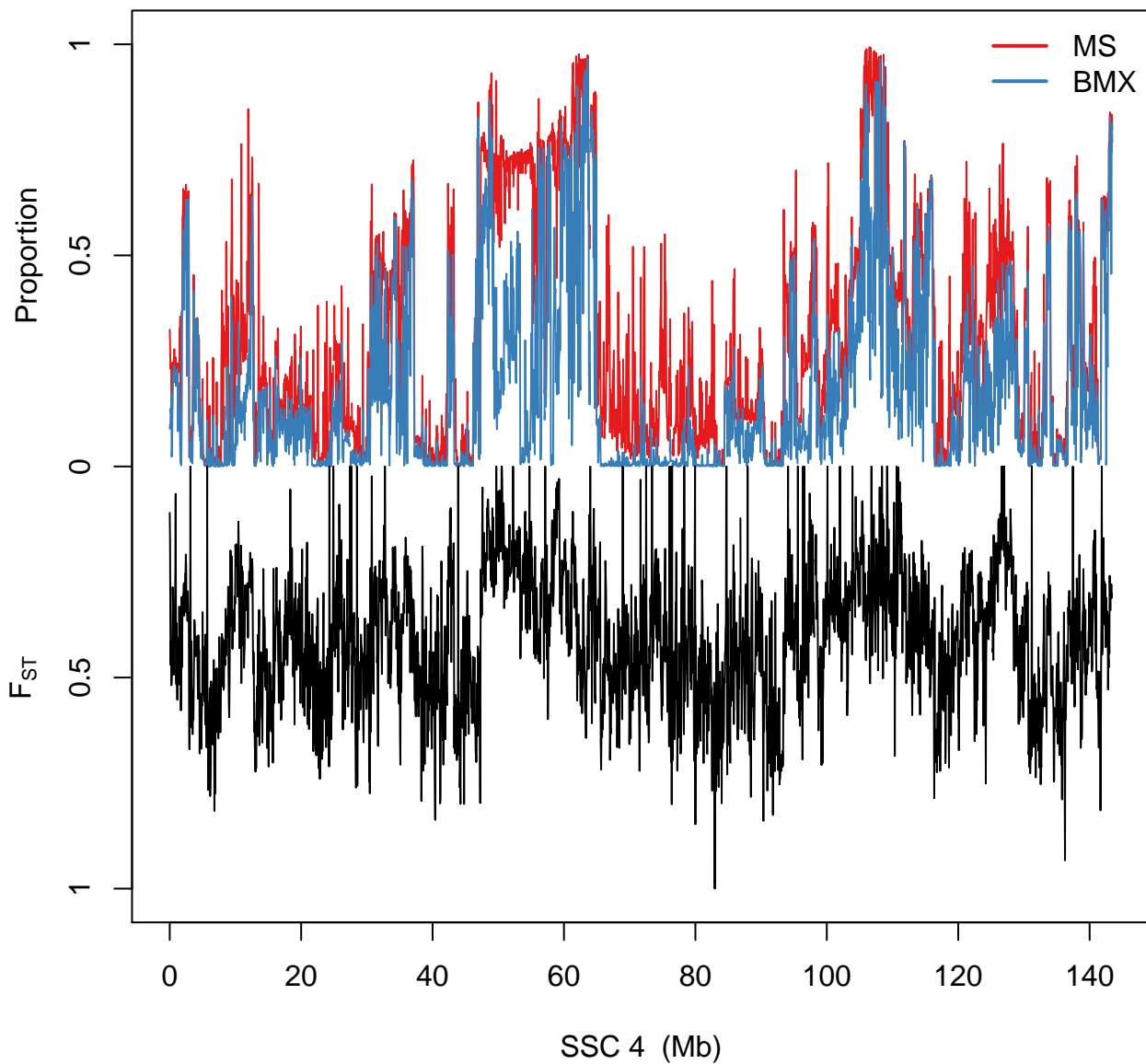

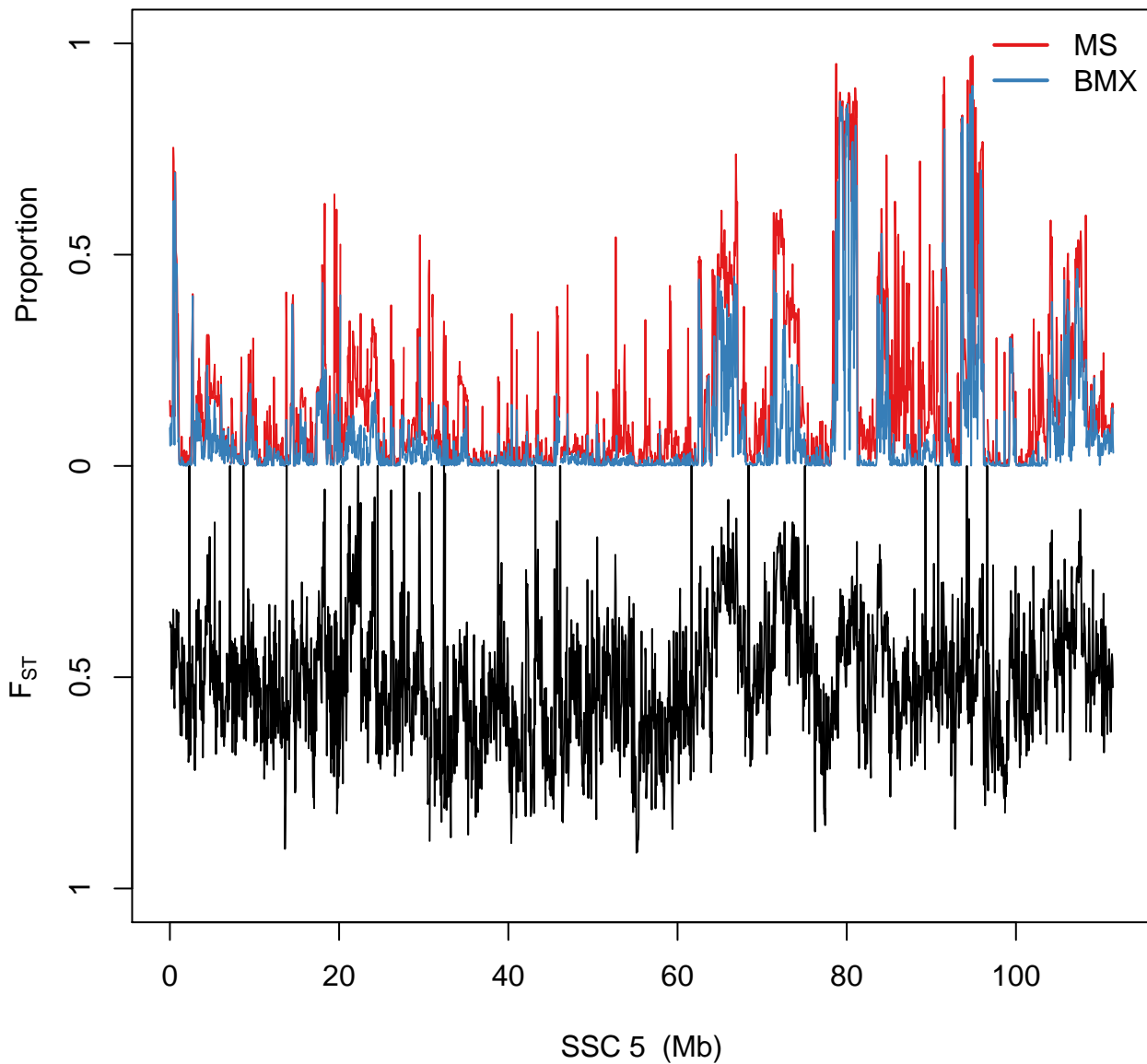

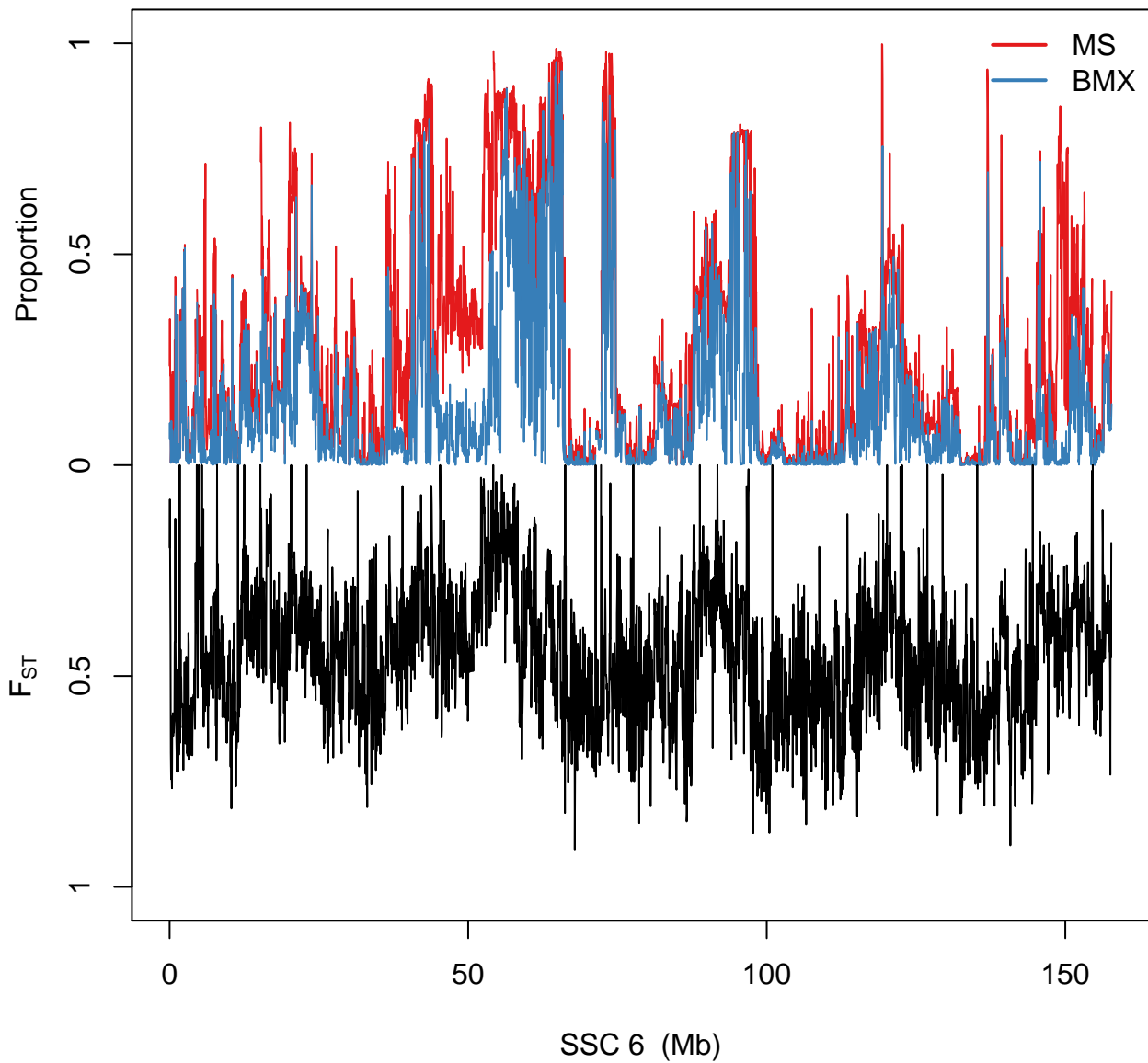

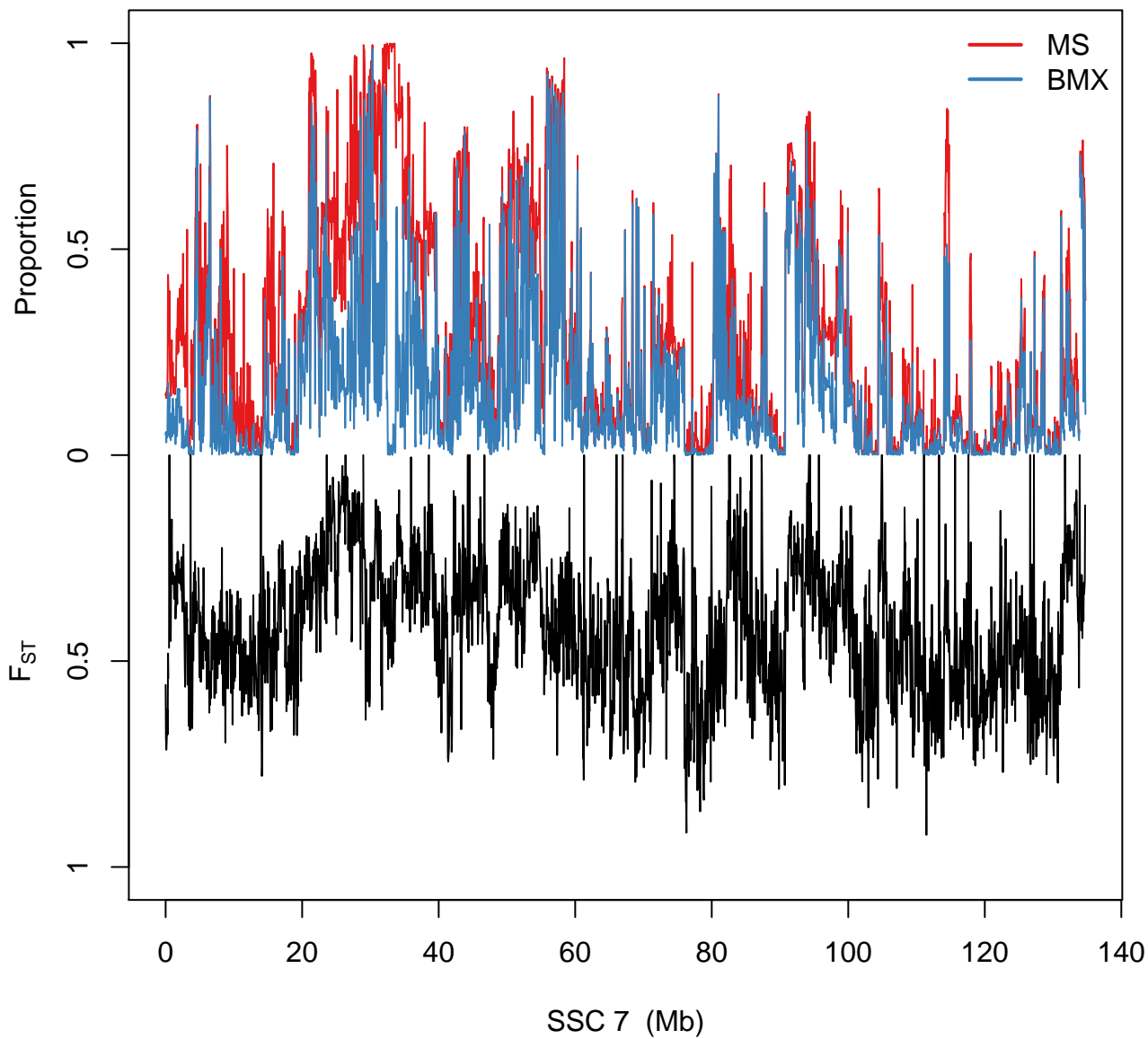

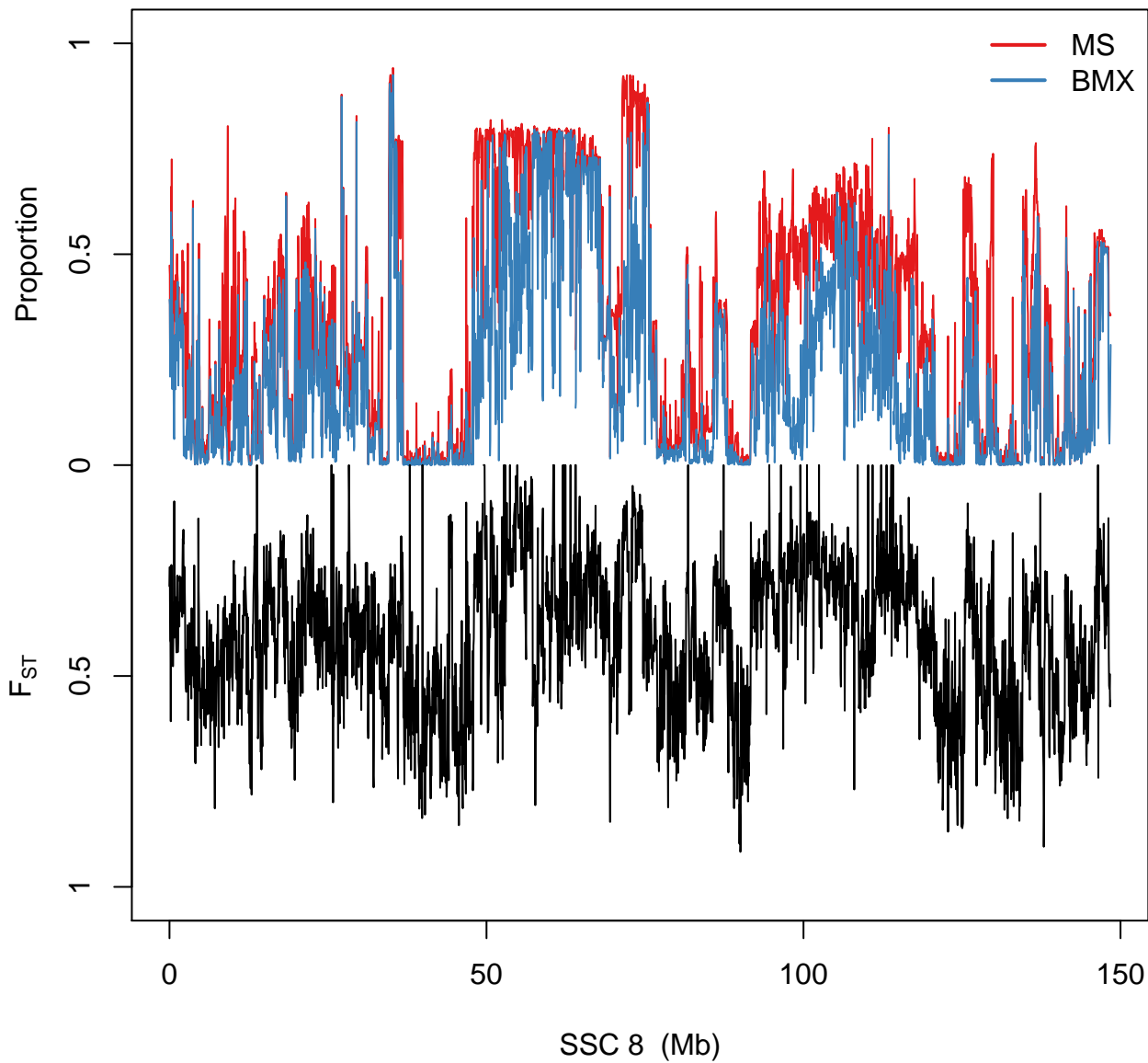

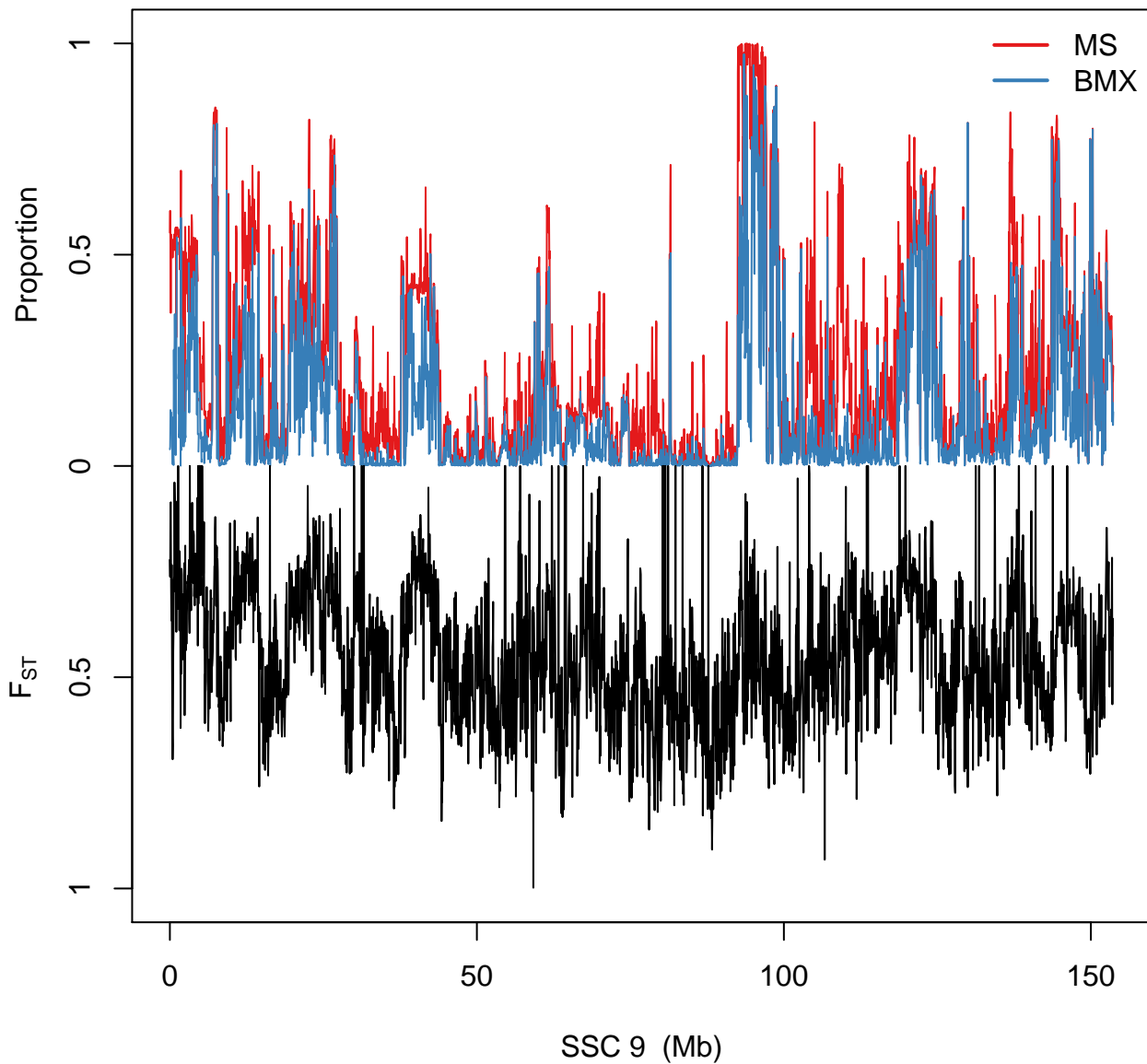

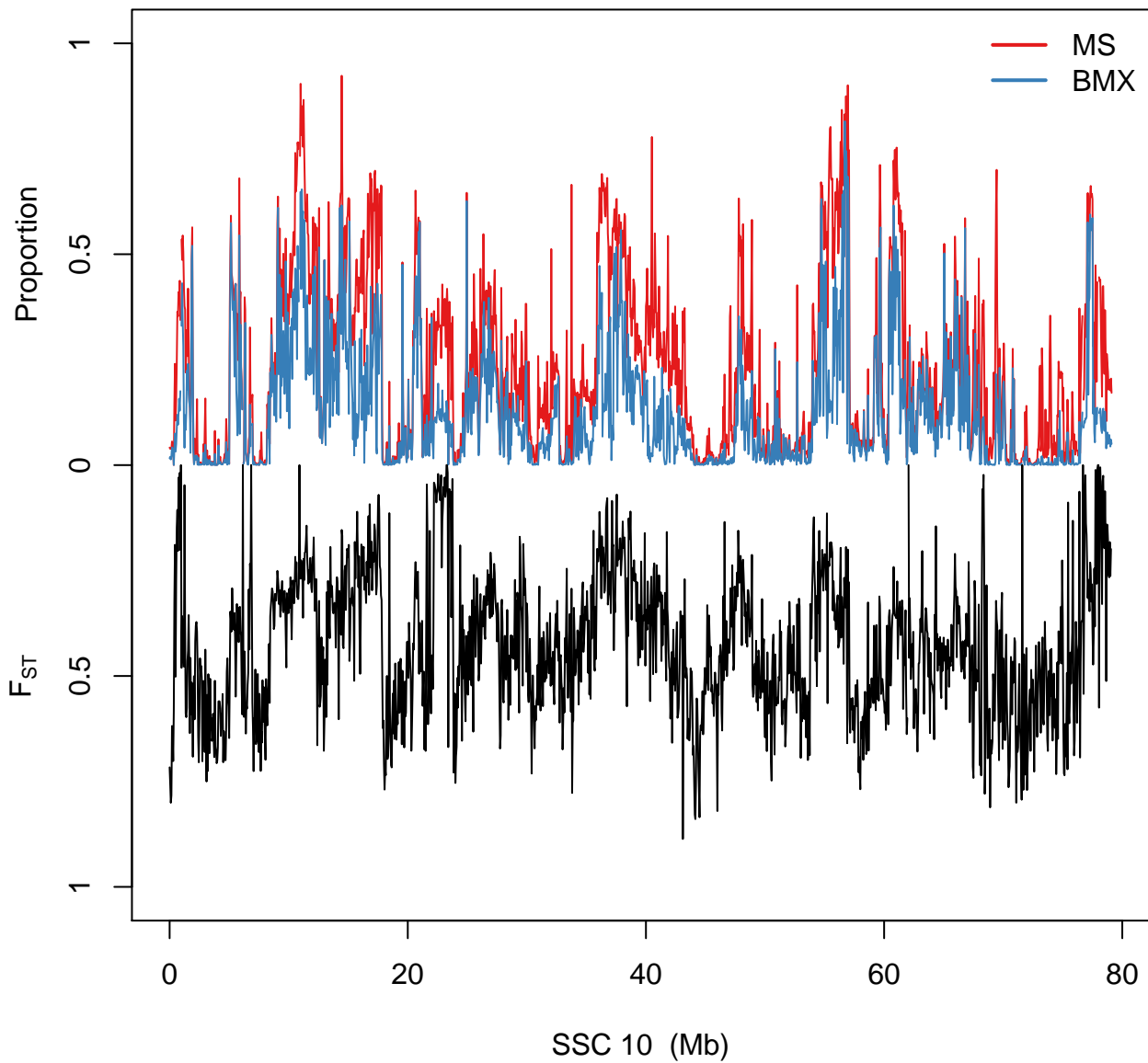

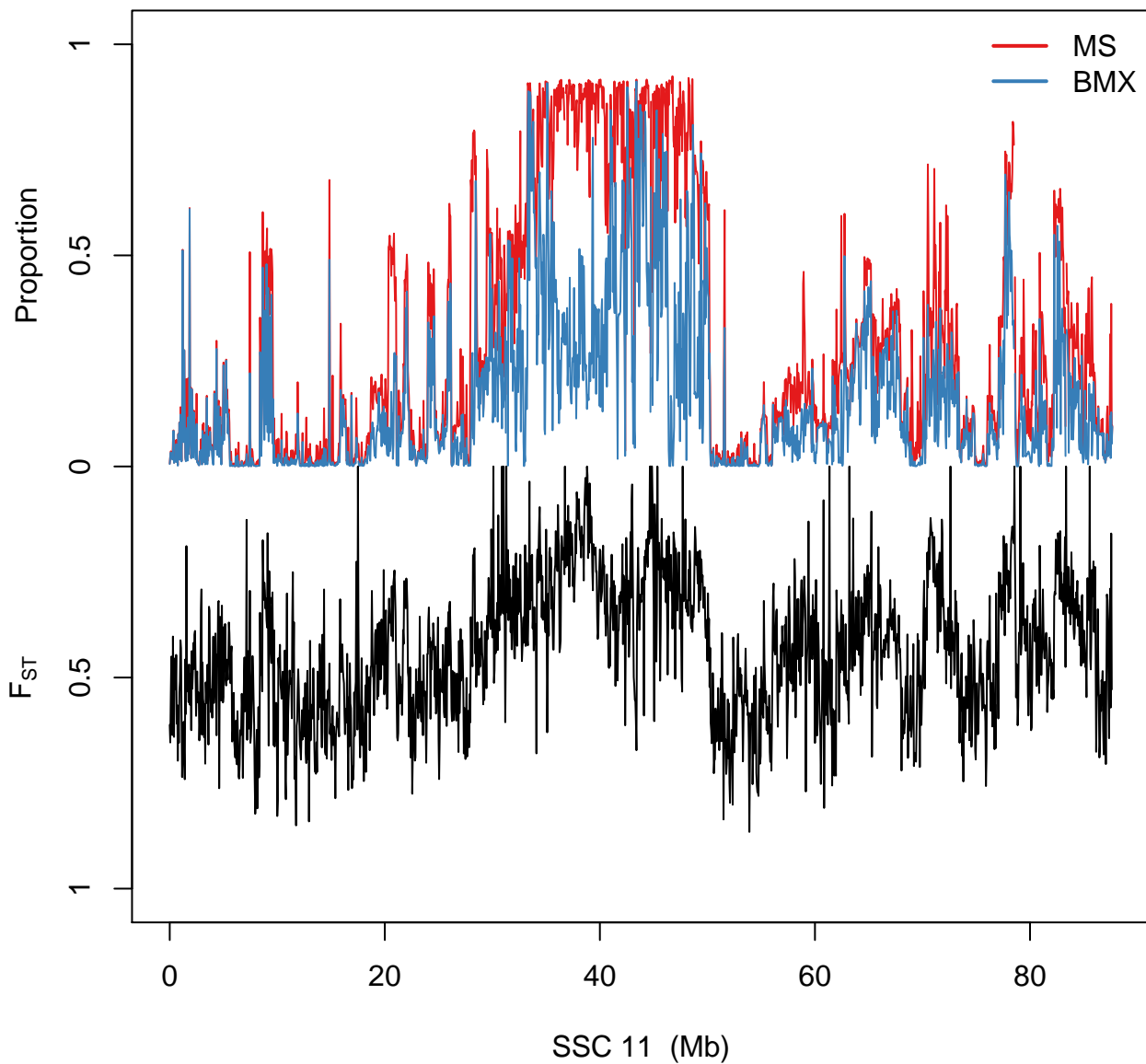

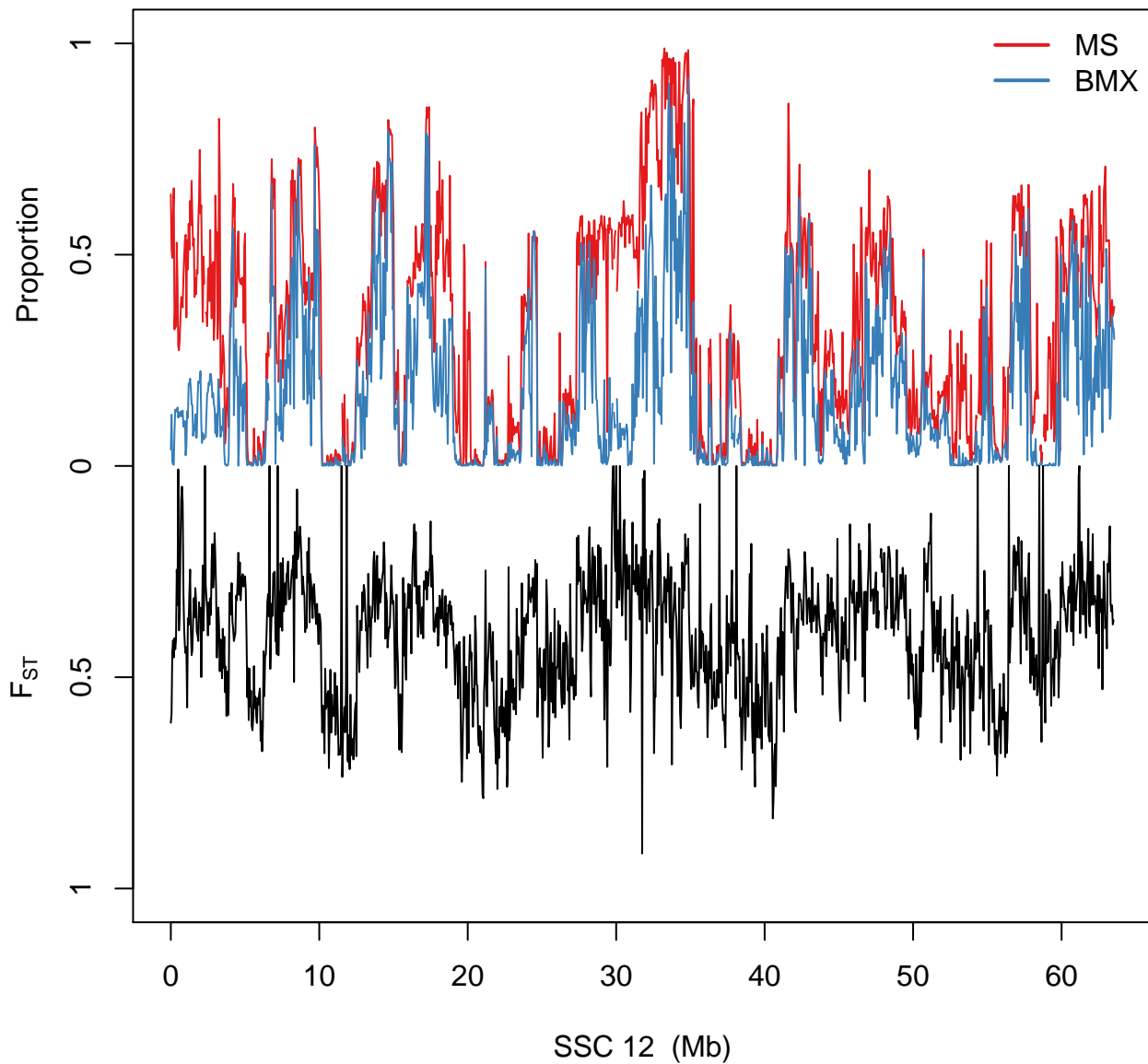

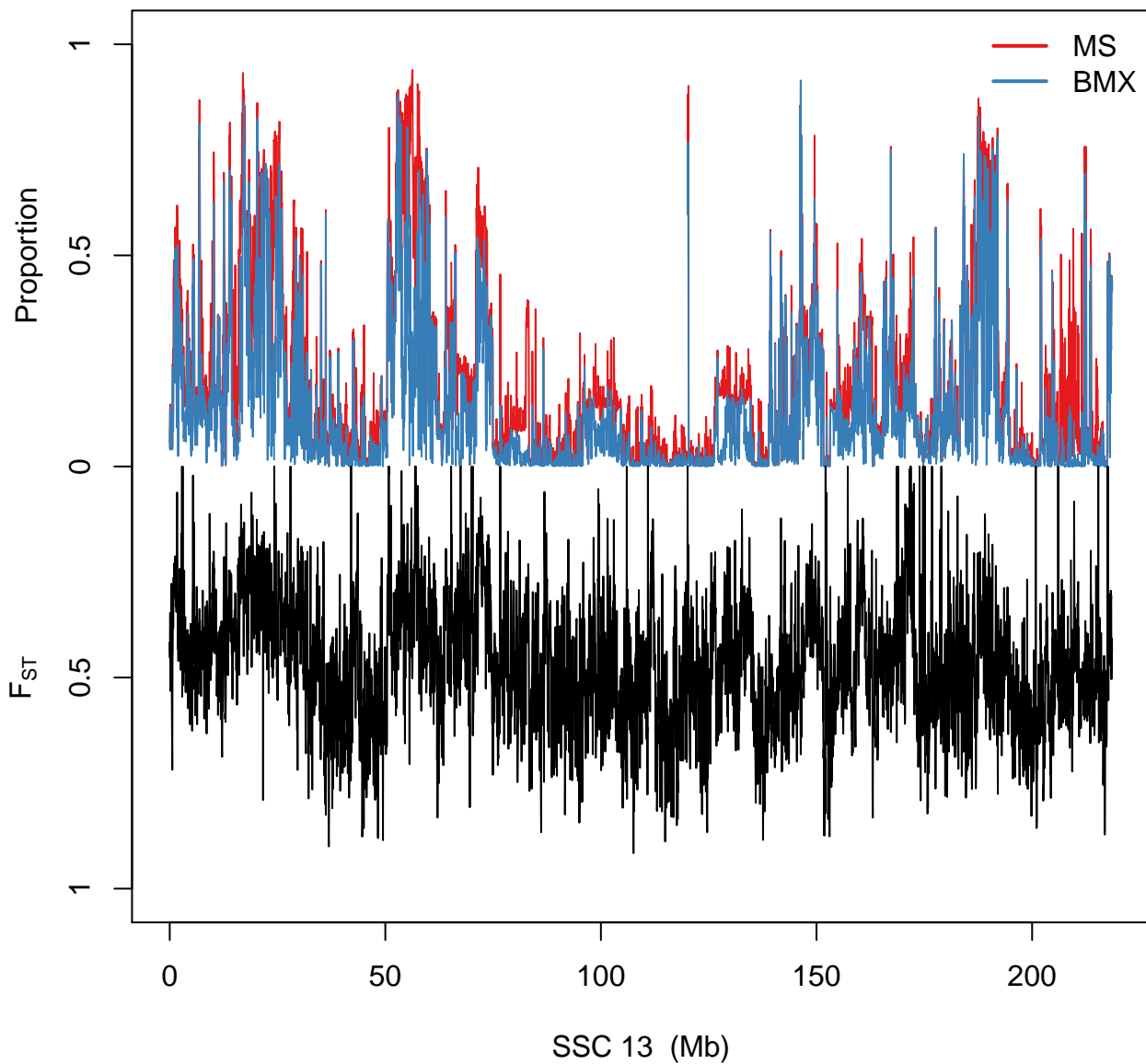

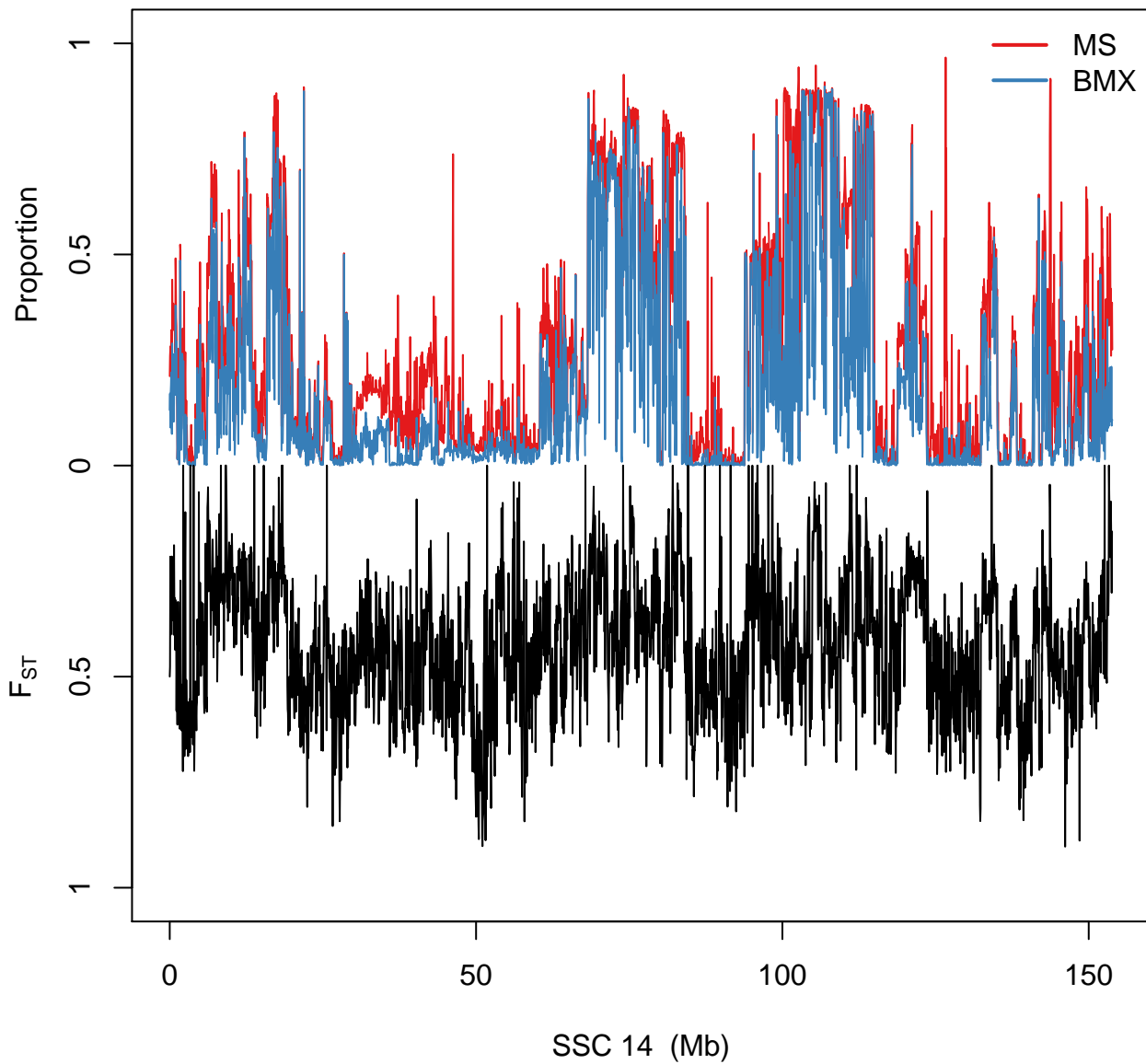

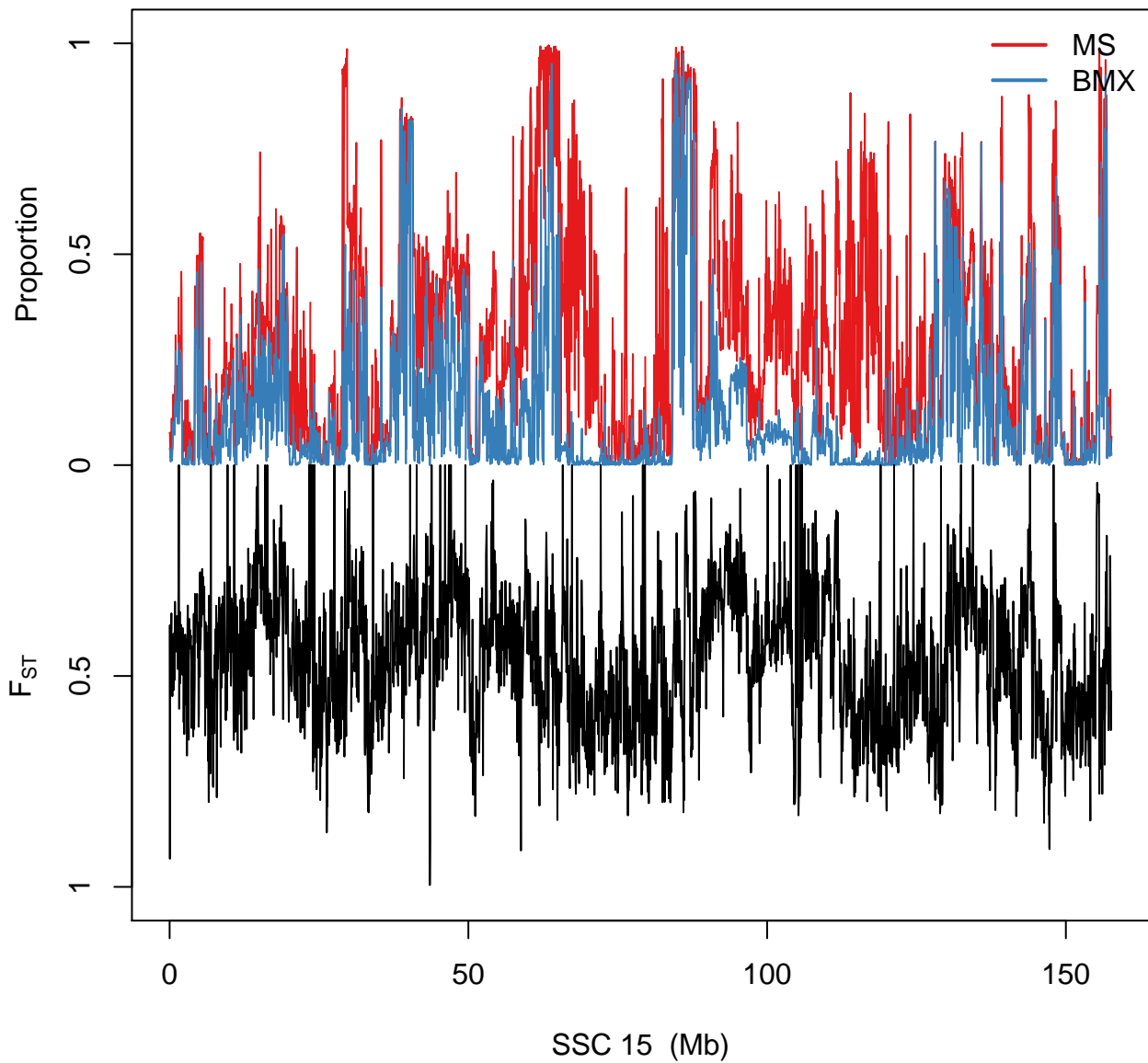

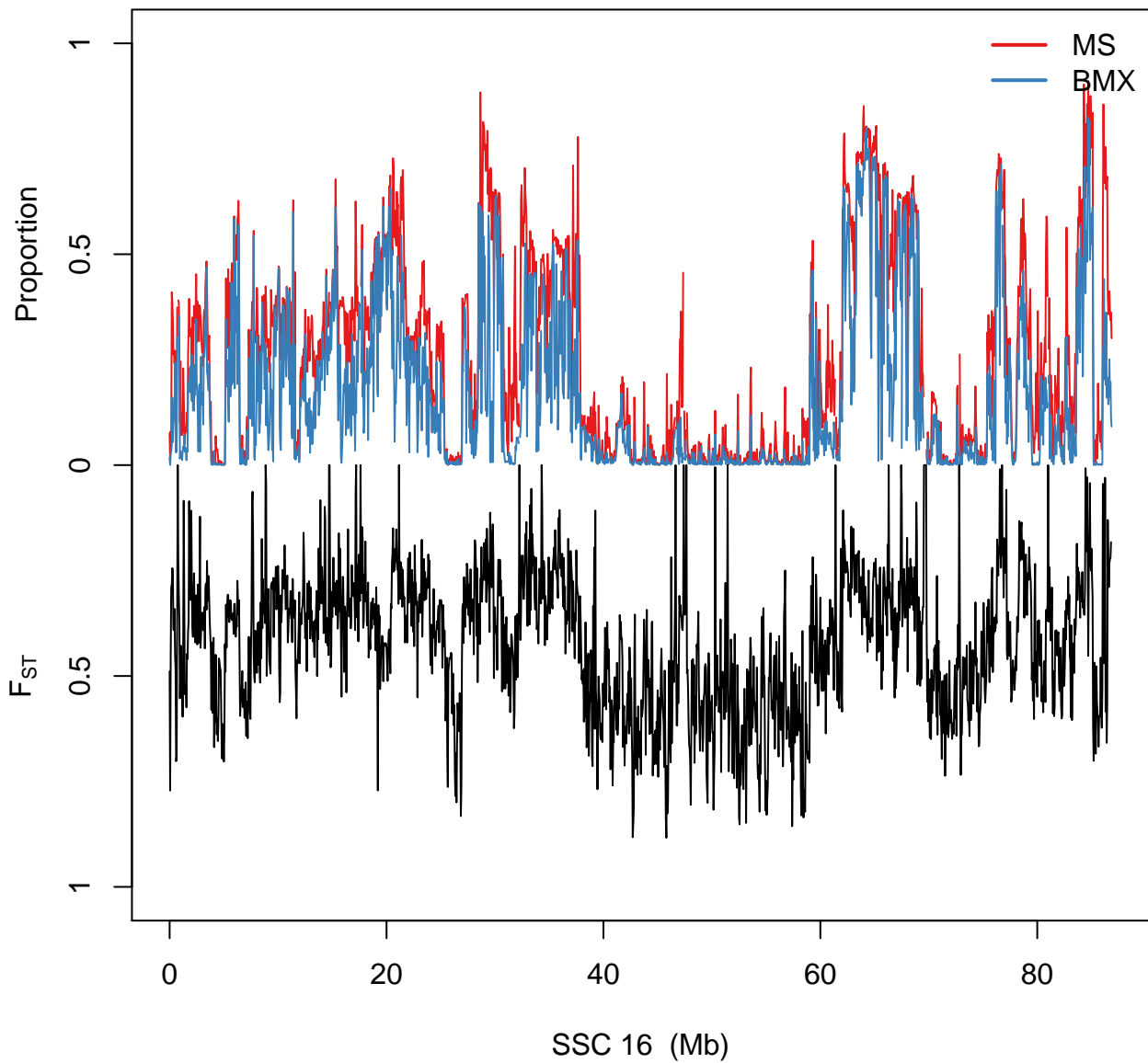

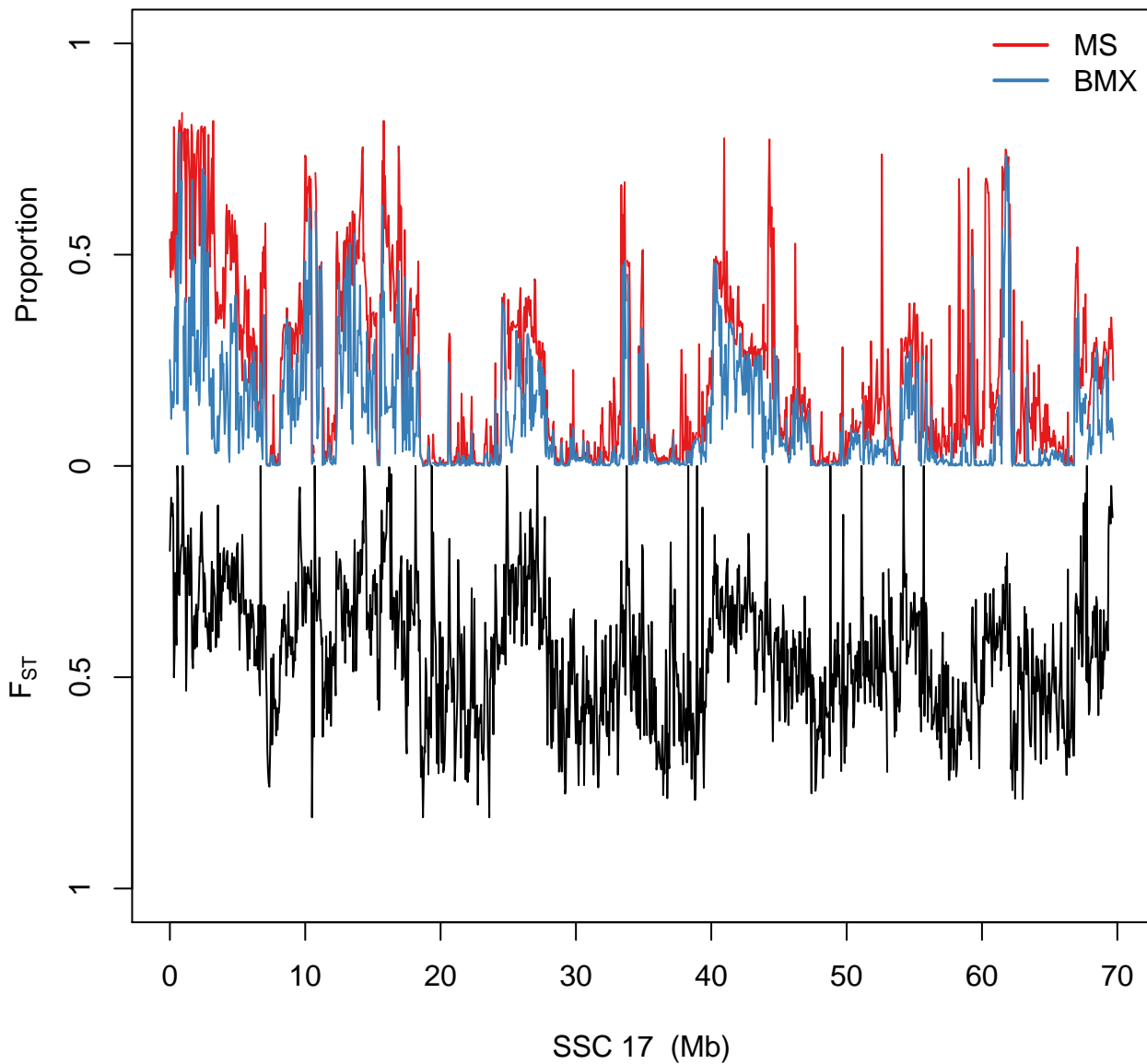

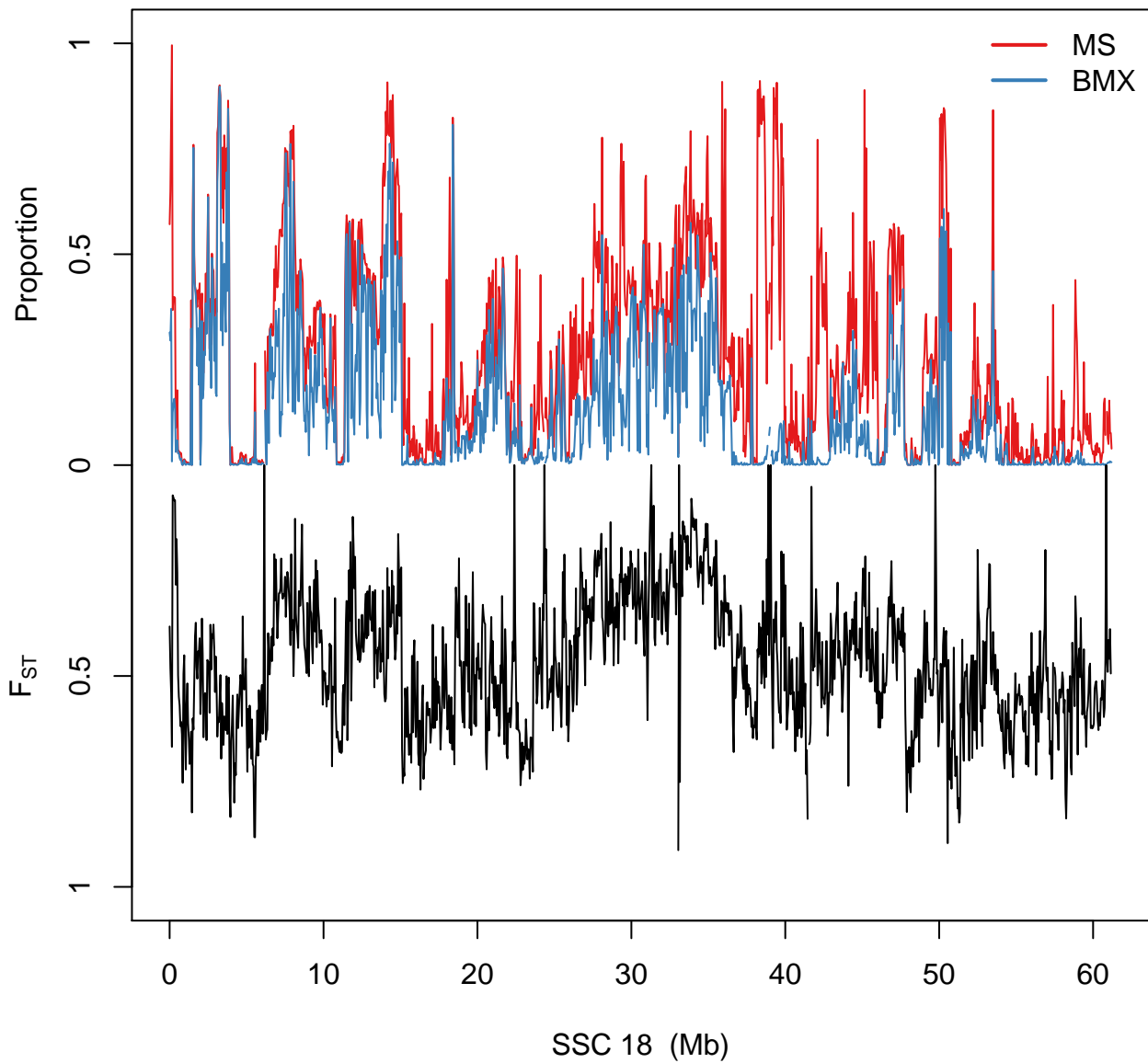

Supplement: Supplementary file 2 [file EVA-12-292-s002.pdf]

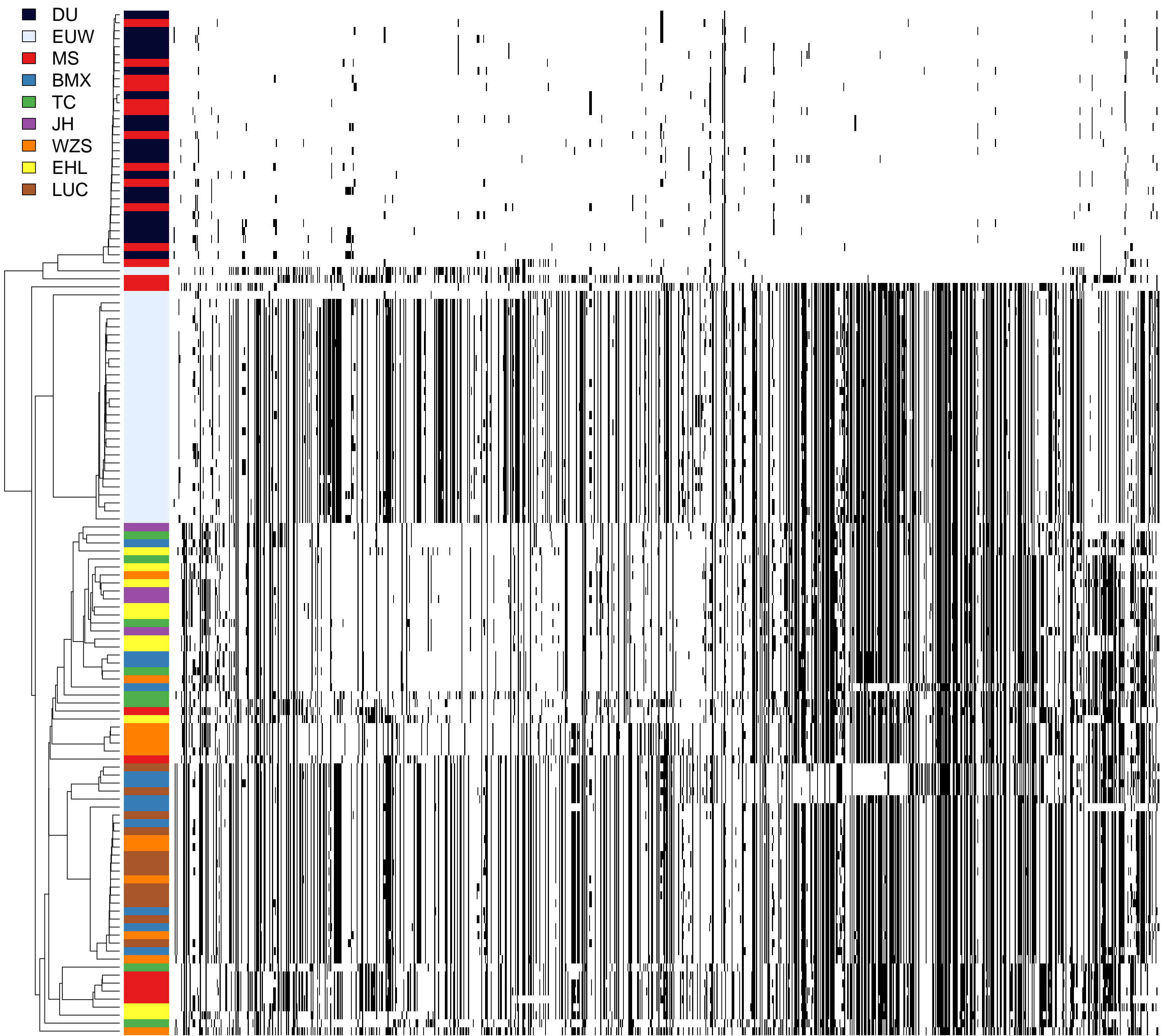

Supplement: Supplementary file 3 [file EVA-12-292-s003.pdf]
